# Advancing Phage Therapy: A Comprehensive Review of the Safety, Efficacy, and Future Prospects for the Targeted Treatment of Bacterial Infections

**DOI:** 10.3390/idr16060092

**Published:** 2024-11-28

**Authors:** Marco Palma, Bowen Qi

**Affiliations:** 1 Institute for Globally Distributed Open Research and Education (IGDORE), 03181 Torrevieja, Spain; 2 R&D Drug Discovery, Protheragen Inc., Holbrook, NY 11741, USA; 3 Drug Discovery and Development, Creative Biolabs Inc., Shirley, NY 11967, USA

**Keywords:** phage therapy, bacteriophage therapy, phage treatment, bacteriophage treatment, phage, bacteriophage, bacterial infection, antibiotic resistance

## Abstract

Background: Phage therapy, a treatment utilizing bacteriophages to combat bacterial infections, is gaining attention as a promising alternative to antibiotics, particularly for managing antibiotic-resistant bacteria. This study aims to provide a comprehensive review of phage therapy by examining its safety, efficacy, influencing factors, future prospects, and regulatory considerations. The study also seeks to identify strategies for optimizing its application and to propose a systematic framework for its clinical implementation. Methods: A comprehensive analysis of preclinical studies, clinical trials, and regulatory frameworks was undertaken to evaluate the therapeutic potential of phage therapy. This included an in-depth assessment of key factors influencing clinical outcomes, such as infection site, phage–host specificity, bacterial burden, and immune response. Additionally, innovative strategies—such as combination therapies, bioengineered phages, and phage cocktails—were explored to enhance efficacy. Critical considerations related to dosing, including inoculum size, multiplicity of infection, therapeutic windows, and personalized medicine approaches, were also examined to optimize treatment outcomes. Results: Phage therapy has demonstrated a favorable safety profile in both preclinical and clinical settings, with minimal adverse effects. Its ability to specifically target harmful bacteria while preserving beneficial microbiota underpins its efficacy in treating a range of infections. However, variable outcomes in some studies highlight the importance of addressing critical factors that influence therapeutic success. Innovative approaches, including combination therapies, bioengineered phages, expanded access to diverse phage banks, phage cocktails, and personalized medicine, hold significant promise for improving efficacy. Optimizing dosing strategies remains a key area for enhancement, with critical considerations including inoculum size, multiplicity of infection, phage kinetics, resistance potential, therapeutic windows, dosing frequency, and patient-specific factors. To support the clinical application of phage therapy, a streamlined four-step guideline has been developed, providing a systematic framework for effective treatment planning and implementation. Conclusion: Phage therapy offers a highly adaptable, targeted, and cost-effective approach to addressing antibiotic-resistant infections. While several critical factors must be thoroughly evaluated to optimize treatment efficacy, there remains significant potential for improvement through innovative strategies and refined methodologies. Although phage therapy has yet to achieve widespread approval in the U.S. and Europe, its accessibility through Expanded Access programs and FDA authorizations for food pathogen control underscores its promise. Established practices in countries such as Poland and Georgia further demonstrate its clinical feasibility. To enable broader adoption, regulatory harmonization and advancements in production, delivery, and quality control will be essential. Notably, the affordability and scalability of phage therapy position it as an especially valuable solution for developing regions grappling with escalating rates of antibiotic resistance.

## 1. Introduction

**Figure 1:**
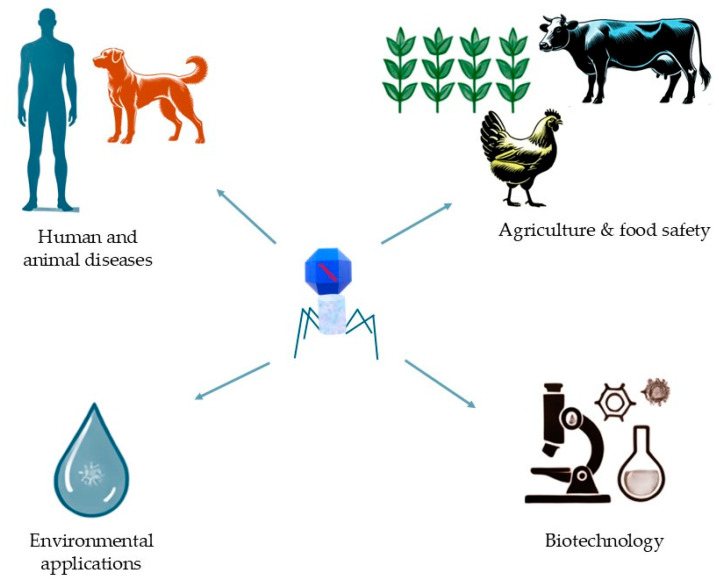
Diverse applications of bacteriophages across sectors. Bacteriophages have broad potential applications, including the treatment of human and animal diseases, enhancement of agriculture and food safety, environmental protection, and various uses in biotechnology.

Bacteriophages, or phages, offer diverse applications across multiple sectors (Figure 1), serving as precise natural agents to control harmful bacteria. In human and animal disease treatment, phages are explored as alternatives to antibiotics, especially against antibiotic-resistant bacteria, due to their ability to selectively target bacterial cells without harming human cells or beneficial microbiota. In agriculture and food safety, phages help control bacterial pathogens in crops, livestock, and food products, reducing foodborne illness risks, minimizing chemical antibiotic use, and promoting environmental safety [1,2]. For environmental management, phages contribute to water treatment, soil health, and sustainable waste processes by targeting harmful bacteria in water sources, soil, and industrial waste, thus reducing the need for chemical disinfectants and aiding in bioremediation [3,4]. In biotechnology, phages are invaluable for gene delivery [5], synthetic biology [6], and protein engineering. Techniques like phage display enable the identification of therapeutic antibodies and immune epitopes, while engineered phages aid in biomanufacturing by controlling bacterial contamination. Through their specificity, phages play crucial roles in advancing sustainable targeted solutions in medicine, agriculture, environmental management, and biotechnology.

Antibiotic resistance presents a significant global health challenge as bacteria increasingly develop resistance to these drugs through their overuse and misuse. This has led to the emergence of bacterial strains, which can resist multiple antibiotics and pose a serious threat to human health. However, there is hope in the form of phage therapy, a potential alternative treatment strategy to antibiotics. Phage therapy involves using bacteriophages or viruses that infect and kill bacteria [7], which are the most abundant organisms in nature [8]. Currently, phage applications in medicine primarily focus on phage-based therapy [9] and phage-based vaccines [10,11].

**Figure 2:**
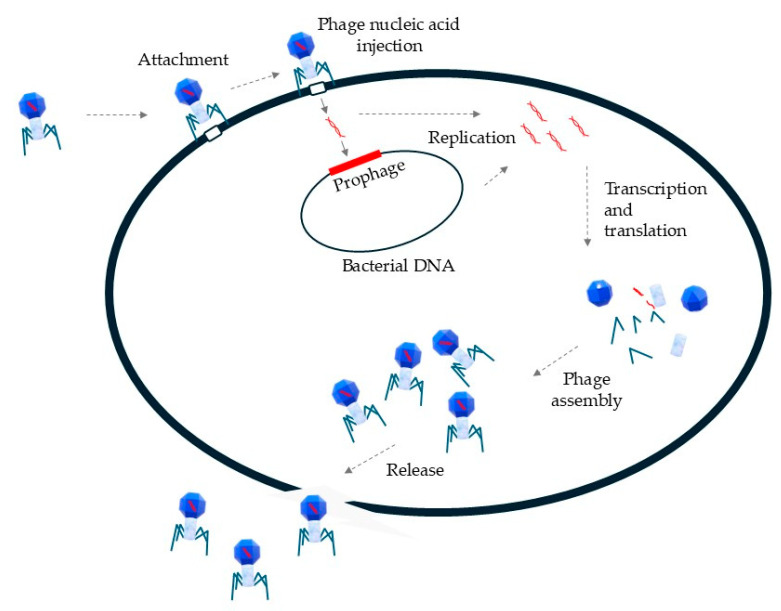
Schematic representation of the bacteriophage life cycle. The bacteriophage life cycle in phage therapy involves several key steps: phage attachment to bacterial cell receptors, the injection of the phage genome, replication and transcription of the phage genome, assembly of new phage particles, and subsequent bacterial cell lysis, resulting in the release of progeny phages.

The mechanism of action for bacteriophages (Figure 2) begins with the recognition and attachment to specific receptors on the bacterial cell surface, allowing precise targeting of particular bacterial strains or species [12]. After attachment, the phage injects its genetic material—either DNA or RNA—into the bacterial cell, leaving the empty capsid outside. Once inside the bacterial cell, the injected phage genome takes over the cell’s machinery to produce essential components, including structural proteins, enzymes, and viral genomes. This replication process can follow either a lysogenic or lytic pathway. In the lysogenic cycle, phage DNA integrates into the host genome, allowing it to be passed to future generations without immediate harm. In the lytic cycle, phage components are produced and assembled into new viral particles. When sufficient particles have formed, phages produce enzymes like endolysins and holins to break down the bacterial cell wall, causing the bacterium to burst (lyse) and release new phages into the environment to infect additional bacteria [13].

Phage therapy has a long history spanning over a century, with popularity in the 1920s and 1930s [14]. However, the advent of antibiotics caused a decline in interest in phage therapy among Western countries, while it continued to be used in the Soviet Union and Poland [7]. Nonetheless, the urgent need to find alternative treatments for bacterial infections has revived interest in bacteriophages as a substitute or supplement to antibiotics [15].

One of the major advantages of phage therapy is its high specificity. Phages can be precisely targeted to kill specific bacteria while leaving beneficial bacteria unharmed in the body. Extensive research has demonstrated the effectiveness of phage therapy in treating a broad range of bacterial infections [16,17,18,19,20] including those caused by antibiotic-resistant bacteria.

Although further research is required to fully understand the potential of phage therapy, it represents a promising alternative to antibiotics and could serve as a vital tool in the fight against antibiotic resistance. This study aims to provide a comprehensive review of phage therapy, shedding light on its potential as a therapeutic option. The review will focus on answering key questions regarding phage therapy: Is it safe for use in both humans and animals? What evidence exists for its effectiveness in preclinical and clinical studies? What are the critical factors influencing the efficacy of phage therapy? What are the future perspectives and potential advancements in the field of phage therapy? Finally, the study will also examine the regulatory landscape surrounding phage therapy.

## 2. Is It Safe for Use in Both Humans and Animals?

Phage therapy has demonstrated a good safety profile in early studies and clinical trials, with minimal adverse effects. One key reason for this is the high specificity of phage therapy, as bacteriophages selectively target and eliminate specific bacteria, sparing beneficial bacteria and eukaryotic cells. This targeted approach reduces the risk of harm to patients and suggests a natural compatibility between phages and humans.

The safety of phage therapy has been evaluated through various administration routes, including oral, local, intravenous (i.v.), and inhalation. When the phage preparation adheres to good manufacturing practices or comparable regulatory standards, only a few adverse events have been reported [21].

Preclinical studies have shown how safe phages are for animals [22,23,24,25], while clinical studies have shown that in humans [26,27,28,29,30,31]. Only mild and temporally symptoms like local reactions at the administration site or transient flu-like were observed in these studies. For example, i.v phage treatments caused occasional flushing or allergies in certain patients [32,33], while intranasal (i.n.) irrigation with phage cocktail resulted in modest side effects like rhinalgia, oropharyngeal discomfort, and metabolic acidosis in other patients [34]. Oral administration of *Escherichia coli* phages caused only transitory stomach discomfort, dyspepsia, and toothache [27]. Additionally, a few individuals developed hypoxemia and hyperthermia during topical pseudomonas phage therapy for burn wounds [35].

A clinical trial assessed the safety of oral administration of the *E. coli* bacteriophage T4. Healthy volunteers between 23 and 54 years of age received high or low doses of T4 or placebo in drinking water at four one-week intervals. Notably, no significant adverse effects were observed, and phages were tolerated well. One week after the 2-day treatment, no fecal phage was detected; however, the overall number of fecal *E. coli* remained unaffected [26].

Another study evaluated the safety of a T4-like bacteriophage cocktail for ColiProteus therapy in Bangladeshi volunteers. The phage formula was administered orally in mineral water three times a day for 2 days. The results showed no adverse effects for more than 3 weeks, even when the bacteriophages were still circulating in the bloodstream. The levels of antibodies against the phages used increased in the blood of the volunteers [27]. These safety results are consistent with a previous study of T4-like phages [28]. Another study demonstrated that coliphage effectively reduced the target organism in the feces of healthy adults and children without impacting the composition of the microbiota [27].

## 3. What Evidence Exists for Phage Therapy Effectiveness in Preclinical and Clinical Studies?

### 3.1. Phage Therapy for Wound Infections

Wound infections are common and usually managed by the body’s natural defenses. However, when these barriers are compromised, serious complications such as bacteremia can occur. Phage therapy offers a promising alternative for treating wound infections, with numerous studies demonstrating its effectiveness and potential as a valuable therapeutic option (Table 1).

*Staphylococcus aureus* is a bacterium that commonly resides on the skin and in the nasal passages of healthy individuals without causing harm [36]. However, if it gains entry into the body through a cut, wound, or other skin breach, it can lead to infections. *S. aureus* is the most frequently identified bacterium in wound infections.

Huon et al. (2020) conducted a study to examine the results of topically applied phages in a mouse model of chronic diabetic wounds infected with *S. aureus*, both when administered alone and in combination with oral amoxicillin-clavulanic acid. The phages PN1815 and PN1957, which were isolated from raw sewage and classified in the families *Myoviridae* and *Podoviridae*, respectively, were used in the study. The phage group received a local application of phage suspension directly on the wound 48 h after bacterial inoculation, either alone or in combination with a 5-day treatment of amoxicillin. Bacteriophage therapy demonstrated improvement in clinical healing and a reduction in local bacterial loads. Surprisingly, the simultaneous administration of phages and antibiotics did not improve the overall survival of the infected mice compared to phage treatment alone [37].

The effectiveness of liposome-entrapped phage cocktails for treating wounds infected with methicillin-resistant *S. aureus* (MRSA) was examined in diabetic mice by Chhibber et al. (2018). The phages (MR-5 and MR-10) used in the phage cocktail were originally isolated from sewage samples. The mice received either liposome-entrapped or non-liposome-entrapped lytic phage cocktails locally, 30 min after the bacterial challenge to the wounds. Mice treated with the bacteriophage cocktail showed a lower wound bioburden and faster tissue repair compared to those receiving a single phage treatment. Notably, a higher phage concentration was detected at the wound site treated with the liposome-entrapped phage cocktail compared to the phage cocktail without liposomes. This indicates that liposome-entrapped phages persist longer at the wound site. The encapsulation of phage mixtures within liposomes presents a promising approach for the treatment of bacterial infections that do not respond to antibiotics [38].

*Pseudomonas aeruginosa* is another common bacterium frequently isolated from wound infections, particularly in hospitalized patients. A preclinical study was conducted in mice to assess the effectiveness of phage treatment in preventing fatal burn wound infections caused by *P. aeruginosa*. An intraperitoneal (i.p.), intramuscular (i.m.), and subcutaneous (s.c.) administration of a phage cocktail containing bacteriophages Pa1, Pa2, and Pa11 was carried out on both infected and uninfected wounded animals. In the absence of phage therapy, the injured mice with infections showed a mortality rate of 94% within the initial 72 h. However, when the phages were injected intramuscularly or subcutaneously, the death rates decreased to 72% and 78%, respectively. Notably, the mortality rate significantly dropped to 12% when the phages were administered intraperitoneally [39].

In another preclinical study, Engeman et al. (2021) analyzed the benefits of combining the phage cocktail PAM2H with antibiotics (ceftazidime, ciprofloxacin, gentamicin, and meropenem) for treating MDR *P. aeruginosa* infections in mice. The phages (EPa5, EPa11, EPa15, EPa22, and EPa43) were previously isolated from sewage. The mice received daily applications of the phage cocktail preparation (25 mL of 1 × 10^8^ Plaque-forming units, PFU) on the infected wound, which was then covered with a Tegaderm™ bandage (3M, St. Paul, MN, USA). The phage formulation was administered alone or in combination with antibiotics, which were given intraperitoneally twice a day. The combination treatment was more effective in eliminating MDR *P. aeruginosa* from wounds compared to either treatment alone [40].

In addition to *S. aureus* and *P. aeruginosa*, phage therapy has also been studied for the treatment of wound infections caused by other bacterial pathogens.

For example, in a preclinical study, researchers assessed the therapeutic effectiveness of a phage formulation combined with a KLY lubricating gel in rats with multi-drug-resistant (MDR) *Klebsiella pneumoniae* wound infections. The phage ZCKP8 (10^9^ PFU/mL), isolated from sewage water, was administered to the infected wounds both with and without the gel 2 h post-challenge. The results demonstrated that phage therapy enhanced wound healing by reducing infection in the treated rats compared to the untreated control group [41].

Khazani Asforooshani et al. (2024) [42] assessed the effect of hydrogel-based *Enterococcus faecium* phage EF-M80 in a wound infection mice model. The phages were isolated from wastewater at Imam Khomeini Hospital in Tehran. In vivo experiments were conducted to examine the therapeutic efficacy of the bacteriophage and evaluate the functionality of the designed hydrogel as a vehicle for delivering the phage to the site of the wound infection. A wound was created on the back skin of the mice, extending below the epidermis and superficial dermis, but without damaging the muscles. All wounds were infected with a suspension of *E. faecium* bacteria. The phages were applied to the wound infection site one-day post-challenge. The wound healing process was monitored over a period of 14 days, revealing a significant improvement in the phage-loaded hydrogel group compared to all other groups. Phage treatment resulted in enhanced wound closure by day 14 in the hydrogel-based *E. faecium* phage group, with noticeable healing starting as early as day 3. The encapsulated phage in the hydrogel demonstrated a synergistic effect, accelerating the wound-healing process. These findings suggest that hydrogel-encapsulated EF-M80 phage could be a promising approach for treating biofilm-related *E. faecium* infections in the future [42].

Karn et al. (2024) conducted a randomized placebo-controlled double-blind clinical trial to evaluate the efficacy of bacteriophage cocktails in treating chronic wound infections caused by multidrug-resistant (MDR) bacteria [16]. The bacteriophages used in the study were isolated from various water sources, including sewage, the river Ganga, ponds, and municipal sewers. The trial included 60 individuals with chronic wounds that had not healed after six weeks of standard therapy and who did not have systemic diseases. Participants were randomly assigned to receive either bacteriophage or placebo treatment. Patients in both the treatment and placebo groups received standard wound care, including debridement, local antiseptics, and local and systemic antibiotics. The wounds were cleansed with sterile saline. In the treatment group, a specific phage cocktail, containing 0.5 × 10^9^ phages per cm^2^ of the wound and targeting the bacteria isolated from each patient, was applied using a sterile gauze piece. The treatment was applied on alternate days for 3 months. The study results demonstrated that 93.3% of the wounds in the phage group achieved sterility within 39 days (median sterility time), with complete healing observed by 90 days. In contrast, 83.3% of patients in the placebo group remained colonized by the original or additional new bacteria, with no healing observed within the same period [16].

Although the aforementioned studies have demonstrated success in using phage therapy for wound infections, other research has not confirmed its efficacy.

For example, Rose et al. (2014) described a clinical trial conducted at the Burn Wound Center of the Queen Astrid Military Hospital in Belgium, which evaluated the safety and efficacy of phage therapy in nine patients with burn wound infections. The phage formula, BFC-1 was a cocktail composed of a pool of 82 phages against *P. aeruginosa* and 8 phages against *S. aureus*, belonging to the phage families Myoviridae, Podoviridae, and Myoviridae, respectively. These phages were sourced from the collections of the Eliava Institute for Bacteriophages. The initial sources of the phages against *P. aeruginosa* were sewage water in Regensburg, Germany, and the Mtvari River in Tbilisi, Georgia. The phages against *S. aureus* are of unknown origin, but the initial place of isolation is Tbilisi, Georgia. The burn wound with a bacterial infection was divided into two sections. One section received standard antimicrobial treatment: amikacin combined with either ceftazidime or meropenem for *P. aeruginosa* infections and vancomycin for *S. aureus* infections. The other section received a phage treatment using BFC-1. This involved applying a single dose of approximately 1 mL of sterile endotoxin-purified BFC-1 per 50 cm^2^ of the wound, delivered with a 5 mL syringe equipped with a spray adapter. Biopsies were taken before the treatments and again two to five hours after the phage treatment. The bacterial loads in all biopsies were measured. Unfortunately, due to the low bacterial burden in most patients both before and after treatment and the small sample size of the study, the authors of this study were unable to determine the efficacy of the BFC-1 phage cocktail [43].

A randomized controlled trial conducted by Jault et al. (2018) investigated the efficacy of phages against *P. aeruginosa* in patients with burn wounds (ClinicalTrials.gov, NCT02116010; European Clinical Trials database, 2014-000714-65). In this study, the researchers used the phage cocktail PP1131, which consists of 12 natural lytic anti-*P. aeruginosa* bacteriophages collected from hospital sewage water. The phage solution was prepared in isotonic saline to achieve a phage titer of 1 × 10^6^ PFU/mL. The treatment involved applying an alginate template impregnated with the phage solution, 20 mL of phage solution for every 200 cm^2^ of algosteril, directly onto the wounds. Another group of patients received Sulfadiazine silver as the standard care treatment. These treatments were administered daily for 7 days, and the patients were observed for 21 days, including the treatment period. The trial was discontinued in 2017 due to the insufficient efficacy of the phage cocktail PP1131 compared to standard care, despite achieving the primary endpoint [35]. The authors noted several limitations and unexpected difficulties during the study, including a small number of participants and stability issues with the phage stocks. Additionally, the standard care treatment was applied directly to the wound, whereas the phage cocktail was administered using alginate templates [35].

The direct application of the phage solution to the wound, without the use of alginate templates, might be a viable alternative.

**Table 1:** Phage therapy for wound infections.

Phage	Origin	Challenge Organism	Bacterial Inoculum (CFU)	Phage Inoculum (PFU)	Delivery Method	Delivery Schedule	Type of Model	Outcome	Reference
PN1815 and PN1957	Sewage	*S. aureus*	10^8^	0.2 × 10^5^	Local	48 h post-challenge	Mouse	Reduced bacterial load and improved healing	Huon et al. (2020) [37]
Cocktails (MR-5 and MR-10)	Sewage	MRSA	10^8^, 10^9^, and 10^10^ CFU/mL	10^9^	Local	30 min post-challenge	Diabetic Mouse	Decreased wound bioburden and improved tissue repear	Chhibber et al. (2018) [38]
Cocktail (Pa1, Pa2, and Pa11)	Sewage	*P. aeruginosa*	10^2^	3.0 × 10^8^	i.p., i.m., and s.c.	Immediately after the bacterial challenge	Mouse	Decreased mortality in mice due to thermal injury	McVay et al. (2007) [39]
Cocktail PAM2H (EPa5, EPa11, EPa15, EPa22, and EPa43)	Sewage	*P. aeruginosa*	10^7^	10^8^	Local with Tegaderm bandage	4 h post-challenge, then once daily on days 1–3	Mouse	The combination treatment improved the elimination of MDR *P. aeruginosa*	Engeman et al. (2021) [40]
ZCKP8	Sewage	MDR *K. pneumoniae*	1.5 × 10^8^ CFU/mL	10^9^ PFU/mL	Local	2 h post-challenge	Rat	Reduced the infection and improved the wound healing	Fayez et al. (2021) [41]
Phage EF-M80	Wastewater	*Enterococcus faecium*	10^7^	10^6^	Local	One day post-challenge	Mouse	Improved wound healing	Khazani Asforooshani et al. (2024) [42]
Cocktails	Various water sources	MDR bacteria	N/A	0.5 × 10^9^ phages per cm^2^ saoked in sterile gauge piece	Local	On alternate days during 3 months	Human, clinical trial	93.3% of the wounds achieved sterility within 39 days, with complete healing observed by 90 days.	Karn et al. (2024) [16]
Cocktail BFC-1 (82 phages against *P. aeruginosa,* and 8 phage against *S. aureus)*	Sewage, Mtvari river, and some of unknow origin	MDR *P. aeruginosa* or *S. aureus*	N/A	10^7^ (per cm^2^ of wound)	Local	Single dose for 2 to 5 h	Human, clinical trial	Unconclusivee due to low bacterial burden before the treatment	Rose et al. (2014) [43]
Cocktail PP1131	Sewage	*P. aeruginosa*	N/A	2 × 10^7^ per cm^2^ algosteril	Local	Daily for 7 days	Human, clinical trial	Lack efficacy	Jault et al. (2019) [35]

CFU, Colony-forming unit; PFU, Plaque-forming units; N/A, Not applicable; i.p., intraperitoneal; i.m., intramuscular; s.c., subcutaneous.

### 3.2. Phage Therapy for Gastrointestinal Infections

Gastrointestinal infections caused by bacteria, commonly referred to as bacterial gastroenteritis, are a global health problem affecting the stomach or intestines and often resulting in diarrhea. Most gastrointestinal infections are not serious and resolve without treatment after a few days. However, in certain populations, diarrheal diseases can lead to significant morbidity and mortality. The elderly, young children, and individuals with chronic illnesses or compromised immune systems can become acutely dehydrated and may require medical attention [44]. Phage therapy offers a promising alternative to antibiotics for treating these types of infections (Table 2).

*Escherichia coli* is the most prevalent bacterium responsible for gastrointestinal infections.

Mao et al. (2023) studied the impact of microencapsulated phage on treating *E. coli* infections in weaned piglets [45]. The study aimed to protect the bacteriophage from the harsh gastric environment, including acidity and proteolytic activity, to enhance the effectiveness of oral phage therapy. The phage (A211) used in the study was isolated from pig farm sewage and microencapsulated using sodium alginate. In the animal model, weaned piglets were orally infected with 3 × 10^6^ Colony-forming units (CFUs) of *E. coli* GXXW-1103 per day from days 2 to 5. Following the bacterial challenge period, the animals received oral phage treatment (5 × 10^9^ PFU) once a day for 7 days. A control group was treated with the antibiotic Florfenicol (FFC). The protective effect of the phage was evaluated by monitoring body weight, assessing bacterial load, and examining histopathological changes. The results showed that phage A221 significantly improved the daily weight gain of piglets, reduced bacterial load in tissues, and alleviated intestinal lesions. Notably, the therapeutic effect of phage A221 was comparable to that of FFC.

Javaudin et al. (2021) explored the effects of phage therapy on the intestinal carriage of multidrug-resistant *E. coli* in a murine model [46]. The study utilized a phage cocktail composed of four lytic phages (PEC02, PEC08, PEC16, and PEC18) that exhibited in vitro activity against an extended-spectrum beta-lactamase (ESBL)-producing *E. coli* strain. To establish a murine model of intestinal colonization, the researchers induced intestinal dysbiosis by orally administering amoxicillin and/or pantoprazole for 8 or 16 days. Seven days after the initiation of this treatment, the mice were challenged with 10⁶ CFU of *E. coli* delivered via 20-GA plastic feeding tubes. Phage therapy was administered either orally or rectally from days 14 to 18, using both encapsulated (10⁸ PFU) and non-encapsulated (10⁶ PFU) phage formulations. Stool samples were collected at multiple time points (1, 6, 8, 10, 14, and 16 days post-challenge) to monitor bacterial concentrations. The findings revealed that oral administration of the phage cocktail in drinking water led to a temporary reduction in fecal concentrations of ESBL-producing *E. coli* 9 days post-challenge. However, the study did not demonstrate the long-term efficacy of phage therapy in reducing *E. coli* carriage.

In a randomized clinical trial (clinicaltrials.gov, NCT00937274), children hospitalized with acute *E. coli* diarrhea at the Dhaka Hospital of the International Centre for Diarrheal Disease Research in Bangladesh received phage therapy. The treatment compositions included either a cocktail of T4-like coliphages (AB2, 4, 6, 11, 46, 50, 55; JS34, 37, 98, D1.4) from Nestlé Research Centre, a commercial Russian coliphage product (Microgen ColiProteus), or a placebo. T4-like *E. coli* phages from the Nestlé Phage Collection were isolated from the stools of children hospitalized with acute diarrhea at the International Centre for Diarrheal Diseases Research in Dhaka/Bangladesh. Microgen ColiProteus is a phage cocktail composed of T7- and T4-like phages; however, their original sources are unclear. The dosing schedule was 1.4 × 10^9^ PFU of the Microgen ColiProteus cocktail or 3.6 × 10^8^ PFU of the T4-like coliphage cocktail, in addition to standard treatment, given orally over 4 days. The results showed no adverse effects caused by the oral administration of the phage composition. However, the treatment did not improve diarrhea outcomes compared to standard care, leading to the discontinuation of the phage therapy. The researchers speculated that the lack of efficacy might be attributed to insufficient phage coverage. They concluded that more studies are needed to understand in vivo phage–bacteria interactions [47].

Salmonellosis is a leading cause of acute bacterial gastroenteritis in humans, primarily resulting from the consumption of animal-derived products, particularly from the poultry and pig sectors. Therefore, controlling *Salmonella* at the farm level is crucial [48]. Phage therapy can serve as an alternative or complement to existing measures for controlling *Salmonella* on farms.

The study by Sevilla-Navarro et al. (2018) aimed to evaluate the use of autophages or bacteriophages isolated from the same environment as the pathogen (from the feces of layer hens), in reducing *Salmonella enteritidis* in environmental and fecal samples on a layer farm [49]. They tested samples from various farm surfaces and layer hens’ droppings, verifying the presence of *Salmonella* in laying hen farms. A phage solution (10^9^ pfu/mL) was sprayed twice over the animals and facility. The results showed that surface samples collected before phage usage were positive for *S. enteritidis*; however, they were negative after phage applications. The number of bacteria decreased in the feces of layer hens after applying bacteriophages. The results indicated that the use of phages could be employed not only as a preventive or prophylactic approach against bacterial contamination in chicken products but also as a complementary technique for cleaning and disinfection.

Zhang et al. (2023) studied how microencapsulation could affect the stability of phages when applied as phage therapy against *Salmonella* colonization in the intestinal tract of chicks [50]. A phage (SP4) specific for *S. Enteritidis* used in this study was isolated from wastewater samples collected from the wastewater treatment station of Hebei University of Engineering in Handan, China. The phages were prepared using a protocol that included xanthan gum, sodium alginate, CaCl_2_, and chitooligosaccharides. Chicks were infected with *S. Enteritidis* by oral gavaging and treated with both free and microencapsulated phages immediately after the bacterial challenge. The chickens were euthanized seven days after receiving the phage treatment. The results indicated a better in vivo therapeutic effect of microencapsulated phages against *Salmonella* infection compared to no treatment or treatment with non-microencapsulated phages. The authors suggested the need to explore other surface coating methods, but it is clear that encapsulation improves the storage of phages for long periods and provides temperature and gastric stability [50].

*Vibrio cholerae* is a pathogen that causes acute diarrheal infection of the intestines, known as cholera, through the ingestion of contaminated food or water. As a result, it represents a significant public health problem [51].

In an early study, Monsur et al. (1970) investigated the effect of high-titer cholera phages on acute cholera patients (n = 8) at the Pakistan-SEATO Cholera Research Laboratory. These patients were severely dehydrated due to diarrhea, with vibrios present in their stools. They received a typical dosage of 100 mL of a phage preparation per hour, containing 2 × 10^12^ phage particles from a cocktail of four phages (Mukerjee’s group I and group IV phages, phage 326, and phage 268), administered via an orogastric tube until the diarrhea ceased. The study concluded that the numbers of *V. cholerae* were rapidly and drastically reduced. However, the high doses of phages were not as efficient as tetracycline. Nevertheless, phage therapy proved useful as it eliminated vibrios without affecting other bacteria in the intestinal flora and did not cause any side effects in the treated patients [52].

Bhandare et al. (2019) investigated the impact of phage Phi_1 on *V. cholerae* infection using an infant rabbit cholera model. Several phages, isolated either from samples of lake water collected in China or from existing collections, were tested in vitro. However, only phage Phi_1 demonstrated a broader host range against *V. cholerae* compared to the other phages and did not contain integrase sequences, making it suitable for therapy. Phage Phi_1 was administered either 6 h before or 6 h after 2-day-old rabbits were inoculated orally with pathogenic *V. cholerae* O1 via catheter. The animals were observed for signs of infection for one-day post-challenge, and samples were taken from their intestinal tracts for analysis. The phage-treated animals showed no clinical signs of the disease, such as diarrhea, loose stools, or significant cecal fluid accumulation, in contrast to 69% of the untreated control group. Additionally, the number of *V. cholerae* recovered from the intestinal tracts of phage-treated animals was significantly reduced compared to untreated animals [53].

The efficacy of phage therapy has also been explored in treating gastrointestinal infections caused by *Clostridioides difficile*, the leading cause of antibiotic-associated hospital-acquired diarrhea in the United States [54]. A significant drawback of antibiotic treatment for *C. difficile infections* (CDI) is its potential to disrupt the gut microbiota, leading to dysbiosis. This imbalance can result in the reduction or elimination of normal gut commensals, creating an environment that facilitates *C. difficile* colonization [55]. Moreover, antibiotic therapy for CDI is often associated with high recurrence rates and poses a risk for the development of antibiotic resistance [54].

The bacteriophages used in the study belong to the family of myoviruses (phiCDHM1 to phiCDHM6) and siphovirus (phiCDHS1) and were isolated from enriched estuarine samples. The efficacy of these phages was tested in vivo using the Syrian Golden hamster model of acute *C. difficile* infection (CDI) [56]. This model accurately mimicked clinical features of the disease, including toxin-mediated diarrhea and tissue pathology. The hamsters were orally challenged with spores of the *C. difficile* CD105HE1 strain (0.2 mL of 10^4^ CFU/mL) and then treated orally with either single phages or cocktails of phages (0.8 mL of 1 × 10^8^ PFU/mL). The first dose was administered at the time of challenge, followed by additional doses every 8 h until the scheduled endpoint of 36 h. The results showed that phage therapy, with some combinations of the studied phages, delayed the onset of symptoms by 33 h compared to the untreated group. Infected untreated animals reached the experimental endpoint at approximately 55 h post-infection, whereas this was delayed to 87 h with phage treatment. Although the experiment was successful, full protection was not achieved, which is consistent with previous publications [57].

Microbiota acts as a barrier against pathogens; therefore, gut microbiota imbalances can impact human health [58]. Bacteriophages may help maintain that balance.

Gindin et al. (2018) conducted a randomized double-blind placebo-controlled clinical trial (clinicaltrials.gov NCT03269617) to investigate the effects of supplemental bacteriophage consumption. This trial involved 43 healthy participants aged 18 to 65 years who experienced mild to moderate gastrointestinal distress. For 28 days, participants received a daily oral dose of a commercial phage cocktail known as PreforPro^®^, which consists of four distinct bacteriophages (LH01-Myoviridae, LL5-Siphoviridae, T4D-Myoviridae, and LL12-Myoviridae). The phage treatment involved applying 10 ng of phage per person per day. The phages were delivered in an inert carrier consisting of rice maltodextrin and coconut oil triglycerides encapsulated in capsules. The main outcomes assessed were a detailed metabolic panel and a digestive health questionnaire. Furthermore, specimens were gathered for subsequent analysis of secondary outcomes, which included overall microbiota compositions, blood lipids, and indicators of local and systemic inflammation. The results indicated that the phage cocktail was safe and well-tolerated among the participants, with no impact on the comprehensive metabolic panel outcomes due to the phage treatment. The participants in this study reported significant improvements in various symptoms of gastrointestinal distress. The researchers concluded that bacteriophages could be used as a dietary supplement for healthy individuals with mild to moderate gastrointestinal distress without worsening their symptoms [59].

Febvre et al. (2019) conducted a randomized double-blind placebo-controlled crossover intervention trial (clinicaltrials.gov as NCT03269617) to examine the effects of supplemental *E. coli*-specific bacteriophages on gut microbiota and markers of intestinal and systemic inflammation in a group of 43 healthy adults aged 18–65. The treatments involved a daily intake of a four-bacteriophage cocktail (LH01-Myoviridae, LL5-Siphoviridae, T4D-Myoviridae, and LL12-Myoviridae) at a concentration of 10^6^ phages per dose over a 28-day period. Stool and blood samples were collected to analyze inflammatory markers, lipid metabolism, and gut microbiota composition. While phage consumption resulted in reduced fecal *E. coli* loads, there were no significant changes in the gut microbiota, as bacterial taxa remained consistent across treatment groups and time points. Short-chain fatty acid production, inflammatory markers, and lipid metabolism were largely unaffected, though a small but significant decrease in circulating interleukin-4 (IL-4) was noted. Overall, the results suggest that the phages did not disrupt the overall microbiota composition [60].

**Table 2:** Phage therapy for gastrointestinal infections.

Phage	Origin	Challenge Organism	Bacterial Inoculum (CFU)	Phage Inoculum (PFU)	Delivery Method	Treatment Schedule	Type of Model	Outcome	Reference
A211	Pig farm sewage	*E. coli*	3 × 10^6^, encapsulated	5 × 10^9^	Oral	Once a day for 7 days	Piglets	Improved the daily weight gain, reduced bacterial load in tissues, and alleviated intestinal lesions	Mao et al. (2023) [45]
Cocktail (PEC02, PEC08, PEC16, PEC18)	Unknown	*E. coli*	10^6^, encapsulated and non-encapsulated	10^6^ and 10^8^	Oral and rectal	Days 14 to 18	Mouse	Temporary reduction in fecal concentrations of *E. coli*	Javaudin et al. (2021) [46]
Cocktail of T4-like coliphages (AB2, 4, 6, 11, 46, 50, 55; JS34, 37, 98, D1.4) and ColiProteus cocktail	Some are from Feces and others are unkown	*E. coli*	N/A	10^8^ or 10^9^	Oral	Apply during 4 days period	Human, clinical trial	Improved diarrhea outcomes	Sarker et al. (2016) [47]
Lytic authophages	Laying hens’ feces	*S. enteritidis*	N/A	10^9^ PFU/mL	Splay	Apply twice with a 24-h interval in between	Apply on layer farm surfaces and layer hens	Farm facilities after phage therapy were negative for *Salmonella* and decreases in the faces of layer hens	Sevilla-Navarro et al. (2018) [49]
SP4	Wastewater	*S. enteritidis*	3 × 10^10^ CFU/mL, 0.5 mL/chicks	3 × 10^10^ PFU/g, 0.5 g/chicks), microencapsulated	Oral	Immediately after the bacterial challenge	Chicks	A better in vivo therapeutic effect of microencapsulated phages	Zhang et al. (2023) [50]
Cocktail (Mukerjee’s group I and group IV phages, phage 326 and 268)	Different sources	*V. cholerae*	N/A	10^12^	Via an orogastric tube	Until the diarrhoea ceased	Human, clinical trial	Reduced the number of bacteria	Monsur et al. (1970) [52]
Phi_1	Lake water	*V. cholerae*	10^8−9^	10^9^	Oral via 5F catheters	6 h before or 6 h post-challenge	Infant rabbit	Reduced clinical sign of the disease	Bhandare et al. (2019) [53]
Cocktail (phiCDHS1, phiCDHM 1–6)	Estuarine samples	*C. difficile*	2 × 10^3^	10^8^	Oral	At the time of the challenge, then every 8 h until 36 h	Hamsters	Delayed the onset of symptoms	Nale et al. (2016) [56]
Cocktail PreforPro (LH01-Myoviridae, LL5-Siphoviridae, T4D-Myoviridae, and LL12-Myoviridae)	Unknown	Against gastrointestinal distress	N/A	10 ng of phage per person and day. The phages within an inert carrier consisting of rice maltodextrin and coconut oil triglycerides capsules	Oral	One 15 mg capsule per day for 28 days	Human, clinical trial	Safe and well-tolerated. Participants also reported significant improvements in several symptoms of gastrointestinal distress	Gindin et al. (2018) [59]
Cocktail PreforPro (LH01-Myoviridae, LL5-Siphoviridae, T4D-Myoviridae, and LL12-Myoviridae)	Unknown	*E. coli*	N/A	10^6^ per dose	Oral	Daily for a 28 day period	Human, clinical trial	The phages did not disrupt the overall microbiota composition	Febvre et al. (2019) [60]

CFU, Colony-forming unit; PFU, Plaque-forming units; N/A, Not applicable.

### 3.3. Phage Therapy for Pneumonia

Pneumonia is a disease with high morbidity and mortality rates globally, and its incidence is on the rise, particularly among immunocompromised individuals, children, and older adults. The common bacterial pathogens responsible for pneumonia include *Streptococcus pneumoniae*, *S. aureus*, Group A *Streptococcus*, *K. pneumoniae*, *Haemophilus influenzae*, *Moraxella catarrhalis*, anaerobes, and various gram-negative organisms. Phage therapy has been tested in several studies focused on pneumonia (Table 3).

Methicillin-resistant *S. aureus* (MRSA) is an important pathogen in ventilator-associated pneumonia (VAP) [61,62]. 

Prazak et al. (2019) conducted a randomized blinded controlled experimental study to assess the efficacy of phage therapy against MRSA using a male Wistar rat model that mimicked VAP. The treatment protocol involved administering a phage cocktail (2–3 × 10^9^ PFU/mL) composed of phages 2003, 2002, 3A, and K. The cocktail was given intravenously at 2, 12, 24, 48, and 72 h after bacterial challenge. The primary outcome was survival, and the secondary outcomes included bacterial load and histopathological scoring of pneumonia. The results showed that all animals treated with phages survived for at least 12 h after infection, with survival rates increasing from 0% to 58% over 96 h post-challenge. This correlated with reduced bacterial burdens in the lungs and improved histopathological outcomes. The efficacy of phage treatment, in combination with the semisynthetic glycopeptide antibiotic Teicoplanin, was also examined for improving survival rates in MRSA infections. However, the combination therapy did not yield superior results compared to either phage therapy or Teicoplanin alone [63].

*K. pneumoniae* can cause dangerous community-onset and nosocomial infections [64,65]. 

Anand et al. (2020) investigated the efficacy of phages against *K. pneumoniae* infection in a mouse model of pneumonia. They used a novel lytic phage (VTCCBPA43) originally isolated from a water sample collected from the River Ganga, Banaras Ghat in India. The BALB/c mice were initially inoculated with *K. pneumoniae* MTCC109, and two hours later, the phage (10^9^ PFU) was administered intranasally. The animals were sacrificed at different time intervals from 6 h to 14 days post-infection to determine the presence of bacteria as well as lung lesions. The results showed that phage therapy successfully prevented the development of severe pathological lesions in the mice and significantly reduced the bacterial load in their lungs [66].

Aleshkin et al. (2016) developed a phage cocktail composed of eight bacteriophages (SCH1, SCH111, KPV15, KPV811, PA5, PA10, AP22, and AM24) from the *Podoviridae* and *Myoviridae* families, capable of lysing several bacterial species, including *K. pneumoniae*, *S. aureus*, *P. aeruginosa*, and *A. baumannii* [67]. These phages have been isolated from clinical materials and wastewater from Moscow. The safety and efficacy of the phage cocktail were tested in mice. For the safety assessment, the animals received a single abdominal injection of the phage composition (0.5 × 10^8^ PFU) given 24 or 12 h before the bacterial challenge. The infected mice were then quarantined for two weeks to monitor their health. For the efficacy assessment, the therapeutic and prophylactic effects of the phage cocktail were experimentally tested against acute lethal *Klebsiella* infection in mice, compared with ciprofloxacin treatment as the standard treatment. The results showed that the animals treated with the phage cocktail survived the *K. pneumoniae* infection and exhibited no symptoms of acute *Klebsiella* infection 14 days post-challenge. In contrast, the untreated animals died between the second and fifth day of the infection. The efficacy of the phage cocktail was found to be comparable to ciprofloxacin, which is highly effective against *K. pneumoniae* infection [67].

*Acinetobacter baumannii* has emerged as a nosocomial pathogen capable of surviving desiccation, disinfectants, and antimicrobials. Carbapenem-resistant *A. baumannii* increases mortality in hospital-acquired pneumonia and bloodstream infections [68]. 

Tan et al. (2021) described a case report of an 88-year-old Chinese man with hospital-acquired pneumonia caused by carbapenem-resistant *A. baumannii* who was treated with phage therapy. The phage preparation consisted of phage Ab_SZ3, previously isolated from sewage and then propagated using the *A. baumannii* clinical isolates obtained from the patient. The patient received a different dose each day, ranging from 5 × 10^6^ PFU to 5 × 10^10^ PFU, in combination with tigecycline and polymyxin E. Ab_SZ3 was administered using a vibrating mesh nebulizer (Aerogen, Galway, Ireland), tigecycline was given intravenously, and Polymyxin E was delivered by inhalation. The phage therapy was administered for 30 min once daily for the first two days, and then every 12 h for 14 days. The researchers concluded that Ab_SZ3 was safe, resulting in the clearance of *A. baumannii* and clinical improvement in the patient’s lung function [69].

*Klebsiella aerogenes*, previously known as *Enterobacter aerogenes*, is an important opportunistic pathogen in humans, posing a serious threat, especially in healthcare settings. This gram-negative bacterium is particularly concerning in patients requiring mechanical ventilation, where it is frequently associated with infections that are difficult to treat due to its multidrug-resistant (MDR) nature. The presence of MDR *K. aerogenes* in such vulnerable populations underscores the critical need for vigilant infection control measures and the development of effective therapeutic strategies [70].

Cui et al. (2023) investigated the therapeutic effects of a bacteriophage in a mouse pneumonia model of *K. aerogenes* [71]. The bacteriophage used in the study, a lytic phage designated pK4-26, was isolated from sewage at the Children’s Hospital affiliated with the Capital Institute of Pediatricsin China. This phage belongs to the *Podoviridae* family and demonstrated bacterial lytic activity and stability under various environmental conditions, such as changes in temperature. To test the efficacy of pK4-26 against *K. aerogenes*, the minimum lethal dose (MLD) was first determined. In the efficacy study, mice received an intratracheal/endobronchial instillation of the MLD of *K. aerogenes*, combined with intranasal administration of pK4-26. The mice were euthanized at various time points, ranging from 2 h to 7 days post-infection, to monitor bacterial loads in the lungs. The results showed that pK4-26 effectively lysed *K. aerogenes* in vivo, reducing mortality and alleviating pneumonia without causing obvious side effects. This indicates that phage pK4-26 is a promising alternative to antibiotics. It can be used in phage therapy to treat pneumonia caused by multidrug-resistant *K. aerogenes* [71].

Samaee et al. (2023) [17] studied the effects of inhalation phage therapy against secondary bacterial pneumonia in patients with COVID-19. The bacteriophage used in the study were isolated from sewage samples collected at Bou Ali Sina Hospital in Sari, Mazandaran, Iran. From the collected phages, a phage cocktail was developed with specificity to *P. aeruginosa, Acinetobacter*, and MRSA, which are common causes of secondary nosocomial infections, including pneumonia. A double-blind clinical trial was conducted with 60 COVID-19-positive patients who were randomly divided into intervention and control groups. The intervention group received 10 mL of phage suspension every 12 h via a mesh nebulizer for 7 days, while the placebo group received a phage-free solution administered in the same manner. The results indicate a significant difference between the inhalation phage cocktail and the placebo regarding the absence of fever and dyspnea after the treatment period, as well as negative sputum culture results. However, there was no statistical difference in survival rates or duration of stay in the intensive care unit. The researchers concluded that inhalation phage therapy did not show any side effects and can be considered a safe treatment for COVID-19 patients. They recommend conducting more clinical trials with controlled confounding factors to further support these findings [17].

**Table 3:** Phage therapy for pneumonia.

Phage	Origin	Challenge Organism	Bacterial Inoculum (CFU)	Phage Inoculum (PFU)	Delivery Method	Treatment Schedule	Type of Model	Outcome	Reference
Cocktail (2003, 2002, 3A, and K)	Unknown	MRSA	6–8 × 10^9^	2–3 × 10^9^ PFU/mL	i.n.	2, 12, 24, 48, and 72 h post-challenge	Rat	Increased survival	Prazak et al. (2019) [63]
VTCCBPA43	River	*K. pneumoniae*	10^9^	10^9^	i.n.	2 h post-challenge	Mouse	Reduced the bacterial load in their lungs	Anand et al. (2020) [66]
Cocktail (SCH1, SCH111, KPV15, KPV811, PA5, PA10, AP22, and AM24)	Waste water	*K. pneumoniae*	N/A	10^8^	i.p.	24 or 12 h before bacterial challenge.	Mouse	Increased survival and eliminated the symptoms of acute *Klebsiella* infection.	Aleshkin et al. (2016) [67]
Ab_SZ3	Sewage	*A. baumannii*	N/A	5 × 10 PFU to 5 × 10^10^	i.n. with aerosol	Daily the first two days and every 12 h for 14 days, in combination with tigecycline and polymyxin E	Human, case report	Reduced bacterial load, improved patient’s lung function	Tan et al. (2021) [69]
pK4-26	Sewage	*K.aerogenes*	2 × 10^7^	2 × 10^9^	i.n.	at the time of bacterial challenge.	Mouse	Reduced mortality and alleviatedg pneumonia	Cui et al. (2023) [71]
Cocktail	Sewage	*P. aeruginosa*, *Acinetobacter*, and MRSA	N/A	10^13^	i.n. via nebulizer	Every 12 h for 7 days	Human, clinical trial	Reduction of secondary infections and improvement in the outcomes of COVID-19 patients.	Samaee et al. (2023) [17]

CFU, Colony-forming unit; PFU, Plaque-forming units; N/A, Not applicable; i.n., Intranasal; i.p., Intraperitoneal.

### 3.4. Phage Therapy for Urinary Tract Infections

Urinary tract infections (UTIs) are caused by a range of pathogens including *E. coli*, *K. pneumoniae*, *Proteus mirabilis*, *Enterococcus faecalis,* and *Staphylococcus saprophyticus* [72].

Several studies have assessed the efficacy of phage therapy for treating UTIs (Table 4).

Mijbel Ali et al. (2021) tested the efficacy of phage therapy for UTIs caused by *E. coli* using a mouse model. In this model, the bladder mucosa of mice was traumatized by injecting 100 µL of HCl solution into the urinary tract for 45 s. The HCl was then neutralized with KOH and washed with sterile saline using a tuberculin syringe. The urinary bladder was inoculated with uropathogenic *E. coli* via a catheter. Treatment consisted of a single dose of phage PEC80 or a phage cocktail administered transurethrally or intraperitoneally, applied 10 days post-challenge. The cocktail contained 25 phages (PEC3, PEC11, PEC15, PEC16, PEC28, PEC30, PEC36, PEC37, PEC38, PEC44, PEC51, PEC52, PEC55, PEC63, PEC68, PEC78, PEC80, PEC94, PEC102, PEC133, PEC215, PEC301, PEC304, PEC305, and PEC306) with strong activity against uropathogenic *E. coli* isolates. Urine samples were collected daily from day 10 to day 20 post-infection. Mice were then sacrificed, and their bladders and kidneys were homogenized, cultured, and analyzed for uropathogenic *E. coli*. The number of bacteria in each organ was calculated. The results showed that PEC80 alone did not affect the therapy, but both delivery approaches of the cocktail formulation resulted in bacterial eradication [73].

In a case report, Terwilliger et al. (2021) described the clinical safety and efficacy of a bacteriophage cocktail in an immunosuppressed 56-year-old male liver transplant patient with complex recurrent prostate and UTIs caused by extended-spectrum beta-lactamase-producing *E. coli*. The phage cocktail formulation consisted of phages HP3, HP3.1, ES17, and ES19, originally isolated from *E. coli* clinical samples. The patient received two weeks of intravenous phage therapy every 12 h at a dose of 10^9^ PFUs/mL, along with six weeks of intravenous ertapenem. Encouragingly, the phage treatment was well-tolerated, with no reported adverse reactions. Following the initial administration of the phage mixture and ertapenem, the patient exhibited negative urine cultures and had no symptomatic recurrences of urinary tract infections during the 12-week follow-up period after completing the treatment). Taken together, this study suggests that the phage cocktail was suitable for the combinatorial treatment with ertapenem for UTIs caused by extended-spectrum beta-lactamase-producing *E. coli* [18].

Kim et al. (2024) from Locu Bioscience described a phase 2 clinical trial named ELIMINATE (clinicaltrials.gov NCT05488340), which investigated the use of the phage cocktail LBPEC01 to treat female patients with uncomplicated urinary tract infections (uUTIs) and a history of drug-resistant UTIs. LBPEC01 is the first CRISPR-Cas3 genetically enhanced *E. coli*-targeting phage cocktail developed. This study consists of two parts: the first part was for dose regimen selection, and the second part was to determine the efficacy, safety, tolerability, and pharmacokinetics. In part 1, 200 mL of 2 × 10^12^ PFU LBP-EC01 was administered over 2 days by intraurethral (IU) administration via catheters, followed by different doses of LBP-EC01 given intravenously over 3 days. All treatments were administered alongside oral trimethoprim/sulfamethoxazole taken twice daily for 3 days. The results indicated that the LBP-EC01 treatment was safe, with no adverse events, resulting in a rapid reduction in *E. coli* in urine on Day 10, and patients were free of UTI symptoms on Day 10 as well as on Day 34 [19].

Multidrug-resistant *K. pneumoniae* is a clinically significant pathogen, responsible for difficult-to-treat pneumonia, urinary tract infections, and bloodstream infections in hospitalized patients [74].

Qi et al. (2021) presented a case report of a 66-year-old man who had previously undergone unsuccessful antibiotic treatment for a multidrug-resistant *K. pneumoniae* UTI. Phage therapy was subsequently applied. Five bacteriophages (Ф902, ФJD905, ФJD907, ФJD908, and ФJD910), previously isolated from various environmental samples, were combined into different phage cocktails. However, these initial combinations failed to eliminate *K. pneumoniae* from the patient’s urine. Therefore, a phage cocktail containing ФJD902 and ФJD905, both lytic to all previous isolates, was administered for a second round of phage therapy. The patient’s bladder was irrigated with a phage solution (5 × 10^8^ PFU/mL) every 48 h for 2 weeks. The patient underwent clinical examinations, and urine cultures were performed. The results indicated that the ФJD902 and ФJD905 phage cocktail successfully reduced the symptoms of the infection, eliminated the bacteria from the patient’s urine, and improved the patient’s bladder condition [20].

### 3.5. Phage Therapy for Bacteremia

Bacteremia refers to the presence of bacteria in the bloodstream, a condition that can have various clinical implications. Under normal circumstances, the blood is sterile, meaning it is free from microorganisms. However, when bacteria enter the bloodstream, it can result in a spectrum of outcomes, ranging from mild and transient bacteremia often resolved by the body’s immune system to more severe conditions such as sepsis, a life-threatening response to infection. Several studies have evaluated the efficacy of phage therapy for treating bacteremia (Table 5).

*K. pneumoniae* is a well-established opportunistic pathogen capable of causing invasive infections in humans, most notably bacteremia. It is a significant clinical concern, ranking as the second most common cause of gram-negative bacteremia, surpassed only by *E. coli*. The threat posed by *K. pneumoniae* is further amplified by its ability to develop antibiotic resistance, particularly through the production of extended-spectrum β-lactamases and carbapenemases. The prominence of *K. pneumoniae* in these infections highlights the critical need for vigilant monitoring and the development of effective treatment strategies, especially in vulnerable patient populations [75].

Shi et al. (2021) assessed the safety and efficacy of phage therapy in an in vivo model of carbapenem-resistant hypermucoviscous *K. pneumoniae* bacteremia. The phage used in the therapy, kpssk3, was previously isolated from raw sewage from Southwest Hospital in Chongqing, China. First, the absolute lethal dose (LD100) of strain NY03 in mice was determined. For the efficacy study, mice were challenged with CR-HMKP at 2 × LD_100_ to induce bacteremia. Three hours post-challenge, the phage kpssk3 treatment (10⁷ PFU) was administered via intraperitoneal (i.p.) injection, either as a single dose or twice daily. Other antibacterial agents were included in the study for comparison. The treatment was successful, with 100% of the mice treated with kpssk3 (10^7^ PFU) surviving the infection and remaining healthy throughout the study. However, when the dosage was decreased to 10^6^ PFU, only 80% of the mice developed bacteremia. Additionally, no significant changes in the gut microbiota caused by kpssk3 were observed [76].

In a separate study, Hesse et al. (2021) examined the survival outcomes of mice infected with multidrug-resistant *K. pneumoniae* following systemic administration of bacteriophages. First, the optimal bacterial dose was determined by injecting different amounts of *K. pneumoniae* intraperitoneally. The phages used in the study, Pharr (P1) and ϕKpNIH-2 (P2), were previously isolated from sewage. To study the efficacy of the phage treatment, the mice were challenged intraperitoneally to induce bacteremia. Subsequently, at different time intervals (1, 8, or 24 h) post-challenge, the mice received i.p. injections of phages, either individually or in combination. The results indicated that combination phage therapy led to the highest increase in survival rates and the lowest incidence of phage resistance among bacteria recovered from the blood and tissues of the mice. The study demonstrates that phage therapy is effective for the treatment of systemic *K. pneumoniae* infection in a mouse model. However, the researchers emphasized that considerable work is still needed to determine how these results can be effectively translated into a viable treatment for humans [77].

*P. aeruginosa* is one of the most prevalent pathogens linked to healthcare-associated infections. It is frequently resistant to antibiotics, leading to substantial morbidity and mortality, particularly in cases of bacteremia [78].

Vinodkumar et al. (2008) evaluated the ability of bacteriophage preparation to rescue septicemic mice with multidrug-resistant (MDR) *P. aeruginosa* infection. The mice that received MLD of the clinical isolate MDR *P. aeruginosa* YFN-58 died within 2 days. The *P. aeruginosa* phage (CSV-31) used in this study was isolated from raw sewage at a municipal sewage treatment plant. The efficacy study assessed phage therapy and was divided into parts: the part was to determine the effect of the phage dose to help mice survive MDR *P. aeruginosa* bacteremia, and the second assessed the impact of delayed treatment on the outcome. The efficacy study of phage therapy was divided into two parts. The first part aimed to determine the effect of various phage doses on the survival of mice with MDR *P. aeruginosa* bacteremia. The second part assessed the impact of delayed treatment on the outcome. In the dose determination phase, different doses of CSV-31 (ranging from 10^4−9^ PFU) were administered intraperitoneally 45 min after the bacterial challenge. In the delayed treatment phase, the highest dose of CSV-31 was administered to the animals at different time points after the challenge. The animals were observed over a 20-day period to evaluate their health condition. The results showed that 100% of the animals survived the infection when they received higher doses of CSV-31, displaying only minimal signs of illness, such as mild lethargy, within the first 24 h. In contrast, the mice became critically ill, with survival rates dropping to 40% and 60% by day 6 and beyond when lower phage doses were administered. The animals survived the infection and remained healthy from day 6 until the study concluded on day 26 [79].

*Enterococcus faecium* is an opportunistic pathogen recognized for its capacity to colonize humans and a wide variety of animal species. The extensive use of antibiotics in hospitals and agriculture has played a pivotal role in the emergence of vancomycin-resistant *E. faecium*, which has become a significant contributor to hospital-acquired infections [80].

Biswas et al. (2002) conducted a preclinical study to evaluate the efficacy of phage therapy using a vancomycin-resistant *E. faecium* (VRE) bacteremia mouse model. Two phages, ENB6 and C33, isolated from raw sewage at a municipal treatment plant, were tested. One-month-old female BALB/c mice were injected intraperitoneally with the minimum lethal dose (MLD) of *E. faecalis*, isolated from a patient’s fecal sample. The study assessed the efficacy of phage therapy in two separate experiments: the first examined the impact of phage dosage on the ability to rescue mice from VRE bacteremia, and the second evaluated the effect of delayed treatment on the outcome. In the phage dose experiment, the animals received varying doses (10^3−9^ PFU) of a single intraperitoneal (i.p) injection of ENB6, administered 45 min post-challenge. In the delayed treatment experiment, the mice received a single injection of the highest dose of phage at different times post-challenge. The animals were monitored for 20 days to assess their health status. The results demonstrated that the bacteremia was lethal within 48 h of infection. However, a single injection of ENB6 (3 × 10^8^ PFU) was sufficient to protect all the animals from death. Even when treatment was delayed until the animals were moribund, approximately 50% were rescued by a single injection of this phage preparation [81].

*Klebsiella oxytoca* is an opportunistic pathogen that plays a significant role in hospital-acquired infections in adults. Its multiple drug resistance is especially concerning, as it diminishes the effectiveness of commonly used antibiotics [82].

Li et al. (2021) studied the efficacy of phage therapy against *K. oxytoca* using a mouse model of bacteremia. The phage (Phage vB_Kox_ZX8) used in this study was isolated from fecal samples collected from the Nanjing Stomatological Hospital. The mouse model of bacteremia involved i.p. injection of varying amounts of *K. oxytoca* AD3 (10^6–8^) to determine the MLD. To assess phage efficacy, mice were challenged i.p. with bacteria at the MLD and then administered different dosages of vB_Kox_ZX8 one hour after the bacterial challenge. Each animal was observed during the study for survival and weight change, and blood and organs were collected at the end of the study. The mice began to gain weight two days after the phage treatment was administered. The phage therapy resulted in the rescue of 100% of the animals when 5 × 10^7^ phages were used, 66% when 5 × 10^6^ phages were used, and 50% when 5 × 10^5^ phages were used [83].

Genetically modified phages show significant promise for the treatment of bacteremia. Westwater et al. (2003) applied an alternative strategy of genetically modified phages to transmit cell death instructions to bacteria during an infection. To test the concept, they used the M13 phagemid system carrying DNA encoding the toxins Gef and ChpBK, whose expression can be regulated by a LacI/IPTG-regulated promoter. These are toxic proteins that can inhibit cell growth and trigger bacterial apoptosis. Mice were first pretreated with cyclophosphamide by intraperitoneal injection to produce a neutropenic state. They were then challenged with a single intraperitoneal dose of *E. coli* strain ER2738 (10^8^ CFU) to induce transient bacteremia, followed by phage lysates and IPTG. The findings revealed that using phages to deliver the lethal-agent phagemids pGef and pChpBK led to a substantial decrease in circulating bacteria compared to the control group. The study’s researchers illustrated that phage delivery systems hold great promise for managing bacterial infections in both medical and veterinary contexts [84].

**Table 5:** Phage therapy for bacteremia.

Phage	Origin	Challenge Organism	Bacterial Inoculum (CFU)	Phage Inoculum (PFU)	Delivery Method	Treatment Schedule	Type of Model	Outcome	Reference
Kpssk3	Sewage	Arbapenem-resistant hypermucoviscous *K. pneumoniae*	10^7^	10^7^	i.p.	3 h post-challenge	Mouse	Rescued 100% of the mice	Shi et al. (2021) [76]
Pharr (P1) and ϕKpNIH-2 (P2)	Sewage	*K. pneumoniae*	5 × 10^7^	5 × 10^7^	i.p.	1 h post-challenge	Mouse	Rescued 100% of the mice	Hesse et al. (2021) [77]
CSV-31	Sewage	Multidrug-resistant (MDR) *P. aeruginosa*	10^7^	10^4−9^	i.p.	45 min post-challenge	Mouse	Rescued 100% of the animals from the infection	Vinodkumar et al. (2008) [79]
ENB6 and C33	Sewage	vancomycin-resistant *Enterococcus faecium* (VRE)	10^9^	10^3−9^	i.p	45 min post-challenge	Mouse	Rescued mice from VRE bacteremia.	Biswas et al. (2002) [81]
vB_Kox_ZX8	Feces	*Klebsiella oxytoca*	5 × 10^6^	10^5−7^	i.p.	1 h post-challenge	Mouse	Rescued 100% of the animals with 5 × 10^7^ phages	Li et al. (2021) [83]
M13	N/A	*E. coli*	10^8^	2 × 10^9^	i.p.	Within 5 min post-challenge	Mouse	Reduction of bacterial load	Westwater et al. (2003) [84]

CFU, Colony-forming unit; PFU, Plaque-forming units; N/A, Not applicable; i.p., Intraperitoneal.

## 4. Which Factors Affect Phage Therapy Efficacy?

Phage therapy has demonstrated both successes and failures in clinical applications, with outcomes largely influenced by several (e.g., site of infection, phage–host specificity, bacterial burden, phage pharmacokinetics, antibiotic resistance, immune response, and bacteria classification based on their location in the host cells and their cell wall morphology). Understanding these factors could enhance its therapeutic efficacy and clarify its limitations.

### 4.1. Site of Infection

The effectiveness of phage therapy often varies depending on the infection location and microbial environment. For instance, phages may show high efficacy in superficial skin infections due to easier access and direct contact with bacterial targets [85]. Topical phage applications often achieve better localized bacterial clearance, particularly for wound and burn infections, in systemic or internal infections such as respiratory or bloodstream infections; phage distribution is more complex. In lung infections, for example, inhaled phages can encounter natural barriers like mucus and immune cells [86], limiting their access to bacteria in some cases. This variability highlights the need to adapt phage delivery methods to specific infection types for optimal outcomes [87].

### 4.2. Phage–Host Specificity

The specificity of bacteriophages for their host is an important factor in the efficacy of phage therapy because phages commonly exhibit a very narrow range for their hosts, which limits their ability to infect specific types of bacteria and ignores others [88]. This specificity derives from the complex interactions between phage surface receptors and bacterial receptors. Selecting the right phages to target specific bacteria is a fundamental step in phage therapy. Identifying phages that can efficiently recognize and infect target bacteria maximizes therapeutic outcomes. Factors affecting phage-host specificity include bacterial surface receptors, phage recognition mechanisms, and genetic compatibility between phage and host [89]. The ability of a phage to attach to and infect bacteria is contingent upon the presence of specific receptors on the bacterial surface. These receptors include glycolipids such as O- and Vi-antigens, integral membrane proteins like OmpF, BtuB, and TolC, as well as flagella proteins including FliC, FljB, and FliK [90]. Distinct bacterial species and even strains have distinct receptors, which leads to varying sensitivity to phage infection.

With their host bacteria, phages have developed a variety of recognition methods. A tail protein or fiber used by certain phages recognizes and attaches to a particularly specific receptor on the surface of the bacterial cell. This starts a chain of events that lead to the phage attaching, injecting DNA, and then replicating inside the bacterial cell [90]. Some phages may be rely on more complex mechanisms including enzymatic or electrostatic interactions.

Phage–host specificity also depends on genetic compatibility, which enables successful infection and propagation. This compatibility involves the phage’s ability to hijack the host’s cellular machinery, evade bacterial defenses, and replicate its genetic material. Research continues to explore these interactions at the molecular level to refine phage selection, develop effective phage cocktails, and engineer tailored phages capable of targeting specific bacterial strains, including antibiotic-resistant bacteria.

### 4.3. Bacterial Burden

Phage therapy is most effective when the bacterial burden is significantly decreased. High bacterial burden create physical barriers that make it hard for phages to reach and infect their target bacteria [91], which can reduce the treatment’s effectiveness. Therefore, understanding the impact of bacterial burden on phage therapy and implementing strategies to address this issue is vital for maximizing treatment success. Additionally, high bacterial burden contributes to a greater diversity of bacterial strains or species within an infection site. This diversity complicates phage therapy because different strains or species may have varying susceptibility to phage infection. To effectively target and eliminate the diverse bacterial population in high load infections, phage cocktails containing multiple phages with broad or narrow host ranges may be necessary.

To overcome the challenges of high bacterial burden, several strategies can be employed in phage therapy. One approach is to use physical methods or adjunctive therapies to reduce the bacterial burden before administering phages. Techniques such as surgical debridement, irrigation, or antibiotic treatment can be employed to decrease the bacterial burden and create a more favorable environment for phage therapy [92,93]. For example, one treatment regimen included ceftriaxone, a cephalosporin antibiotic that inhibits bacterial cell wall synthesis. While ceftriaxone shows strong in vitro activity against *Y. pestis* strains, it provides limited protection in mouse models of pneumonic plague, resulting in 80% mortality with single treatments. However, combining ceftriaxone with a phage cocktail significantly improved outcomes, achieving 100% survival and the complete clearance of pathogens from internal organs [94].

Another strategy involves optimizing phage delivery methods to enhance their ability to reach the target bacteria [95]. This can include the development of targeted delivery systems, such as encapsulating phages in nanoparticles or incorporating them into gels or creams, which can improve their stability, bioavailability, and tissue penetration. Engineering phages with improved motility or attachment capabilities may also aid in overcoming physical barriers associated with high bacterial loads.

Furthermore, the strategic selection of phages with broader host ranges or the utilization of phage cocktails consisting of multiple phages holds immense potential in improving the chances of successful infection in high-load scenarios. The impressive capacity of broad-host-range phages to infiltrate and subdue a diverse array of bacterial strains or species, combined with the diverse approach offered by phage cocktails, greatly heightens the likelihood of achieving effective treatment outcomes [57,96].

To enhance the effectiveness of phage therapy under high bacterial burden, a comprehensive understanding of the dynamics between phages, bacteria, and the host environment is crucial. Research efforts are focused on elucidating the interplay between phages and biofilms, deciphering the mechanisms of bacterial resistance to phages, and optimizing phage formulations and delivery systems to improve their therapeutic potential.

### 4.4. Pharmacokinetics

Pharmacokinetics studies how a substance is absorbed, distributed, metabolized, and eliminated by the human body. Understanding the pharmacokinetics of phages is crucial to determine their therapeutic efficacy. Several factors come into play when considering the distribution, metabolism, and elimination of phages, all of which impact their overall efficacy [97].

**Figure 3:**
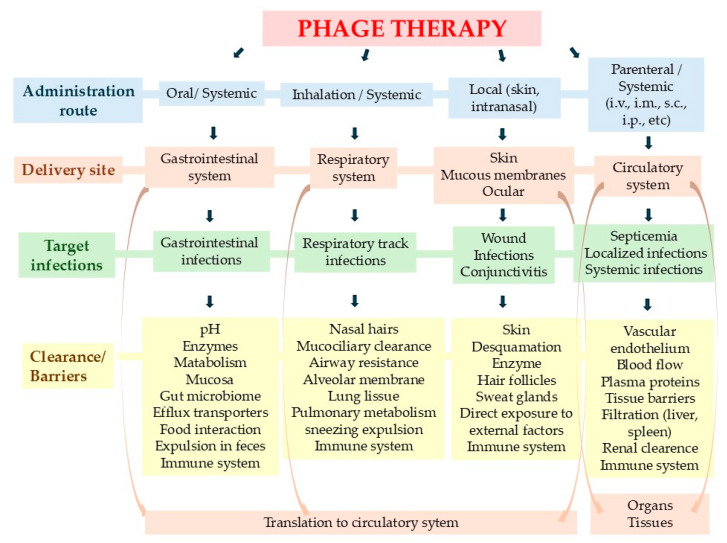
Schematic representation of bacteriophage pharmacokinetics. Illustration of the distribution and clearance of bacteriophages in the body, highlighting how these processes vary based on the chosen route of administration.

Administration routes play a crucial role in the effectiveness of phage therapy (Figure 3) and phage pharmacokinetics [98]. Intravenous (IV) and topical administration enable rapid phage delivery to infection sites, allowing phages to target and attack bacterial cells sooner, which can lead to faster infection control and potentially better clinical outcomes. In contrast, phages administered orally take approximately 2–4 h to appear in the bloodstream [99]. Oral administration also presents challenges, such as inactivation by gastric acid, which can reduce phage efficacy [100].

Phage concentration in vivo is further influenced by dose and treatment frequency [101]. Insufficient dosing can allow bacterial regrowth, whereas optimal dosing schedules have been associated with improved infection control, especially in multi-drug-resistant infections. Achieving an optimal treatment regimen requires striking a balance between maintaining therapeutic phage levels and minimizing potential adverse effects. It is crucial to consider the phage’s specific characteristics, the nature of the target infection, and individual patient factors when determining the most suitable dosage and treatment frequency.

Metabolism plays a vital role in the pharmacokinetics of phages. Metabolic inactivation of phages commonly involves host immunity, such as the phagocytosis of Kupffer cells, low pH inactivation in the stomach, or the production of antibodies by the splenocytes to inactivate phages [97]. Understanding the metabolic pathways can further assist in identifying potential drug interactions or contraindications with other medications or substances that may impact their efficacy or safety.

The elimination of phages from the body is another critical aspect of pharmacokinetics. Unlike many drugs that are renally eliminated, phages exhibit poor renal excretion and great individual variabilities [21,101,102]. Consistent with the preferential biodistribution of phage to the liver and spleen, nonspecific clearance by the mononuclear phagocyte system is likely the primary mechanism for phage elimination from the blood [103].

### 4.5. Antibiotic Resistance

The emergence and spread of antibiotic-resistant bacteria pose significant challenges to phage therapy. Antibiotic resistance developed by bacteria can confer cross-resistance to phages, making them less susceptible to phage infection [104]. Consequently, the effectiveness of phage therapy may be diminished in the presence of antibiotic-resistant bacteria. Understanding the interplay between antibiotic resistance and phage therapy is crucial for developing strategies to overcome this limitation.

One major factor contributing to the reduced efficacy of phage therapy against antibiotic-resistant bacteria is the alteration or loss of bacterial surface receptors that phages rely on for attachment and infection. Antibiotic-resistant strains may undergo genetic changes that modify or eliminate the receptors targeted by phages, effectively blocking their entry into the bacterial cell. This receptor modification can occur through various mechanisms, such as mutation, horizontal gene transfer, or the acquisition of mobile genetic elements, which allow bacteria to rapidly adapt and develop resistance to both antibiotics and phages [105,106]. Additionally, bacteria can employ other defense mechanisms, such as the production of extracellular polysaccharide capsules or biofilms, which can shield them from phage attack. These protective structures act as physical barriers, preventing phages from accessing the bacterial surface and limiting their ability to effectively infect the resistant bacteria. However, certain phages carry phage enzymes capable of breaking down such components [107]. Moreover, the production of bacterial enzymes, such as restriction-modification systems or CRISPR-Cas systems, can provide bacteria with mechanisms to degrade or neutralize phage genetic material, further reducing the efficacy of phage therapy.

To combat antibiotic resistance in phage therapy, several innovative strategies can be pursued. One promising approach involves the identification and isolation of phages specifically tailored to target antibiotic-resistant strains. These phages possess unique receptor recognition mechanisms that can overcome the modifications made by bacteria to evade phage infection [108]. Additionally, the use of phage cocktails, comprising multiple phages with diverse host specificities, increases the likelihood of successful infection by targeting multiple pathways employed by antibiotic-resistant bacteria to resist phages [109].

Another strategy involves the modification or engineering of phages to enhance their effectiveness against antibiotic-resistant bacteria [110]. This can include the genetic modification of phages to encode enzymes that can degrade the protective capsules or biofilms produced by bacteria. Additionally, the development of chimeric phages, combining genetic material from multiple phages, can create hybrid phages with broader host ranges or enhanced infection capabilities. Furthermore, combining phage therapy with other treatment modalities, such as antibiotics or adjuvants, may synergistically enhance the overall effectiveness of treatment against antibiotic-resistant bacteria. Antibiotics can potentially weaken bacterial defense mechanisms, rendering them more susceptible to phage infection. Adjuvants, such as compounds that disrupt biofilms or enhance bacterial membrane permeability, can also be utilized to augment phage access to resistant bacteria. By employing these multifaceted strategies, we can strengthen the arsenal against antibiotic resistance and improve treatment outcomes.

### 4.6. Immune Response

The immune response of the host plays a critical role in shaping the efficacy of phage therapy. While phages are natural enemies of bacteria, they are still foreign entities to the human body, and their presence can trigger immune responses that impact their effectiveness. The immune system has the capacity to neutralize or eliminate phages [111,112,113], thereby diminishing their therapeutic potential. Understanding the complex interplay between phages and the immune response is vital for optimizing phage therapy outcomes. When phages are administered into the body, they can be recognized as foreign antigens by the immune system. This recognition triggers an immune response aimed at clearing the phages from circulation [114]. The immune response can involve the production of neutralizing antibodies, which bind to and inhibit the activity of phages [115], rendering them ineffective against their target bacteria. Furthermore, immune cells, such as phagocytes, can engulf and eliminate phages, preventing their interaction with bacteria and hindering their therapeutic action.

For example, Bernabéu-Gimeno demonstrated that patient serum collected prior to the first phage administration had no effect on phage titer. However, serum collected just 10 days post-administration exhibited neutralizing activity against the administrated bacteriophages, which progressively increased over time and was detectable up to 51 days following phage administration [116].

Additionally, immunocompromised patients may experience more favorable responses [117], as their immune systems are less likely to target phages, whereas immune-competent individuals may have more variable responses. Individual immune status, therefore, need to be considered in tailoring phage therapy regimens for different patient populations.

To mitigate the impact of the immune response on phage therapy, several strategies can be employed. One approach involves the selection of phages with low immunogenicity [118]. By identifying and using phages that are less recognized by the immune system, the likelihood of immune-mediated clearance or neutralization can be reduced, thereby improving the efficacy of phage therapy. Another strategy is to modify or cloak phages to evade immune detection. This can be achieved by coating phages with polymers [119] or modifying their surface properties to make them less recognizable to immune cells and antibodies [120]. These modifications aim to prolong the circulation time of phages in the body and enhance their ability to reach and infect target bacteria before being cleared by the immune system.

It is also important to note that the host immune response can have a dual effect on phage therapy. While an immune response may diminish the effectiveness of phages, it can also contribute to therapeutic outcome by providing an additional layer of defense against bacteria. The immune response can work synergistically with phages to eliminate bacterial pathogens and promote the clearance of infected tissues [114,121]. A study conducted by Roach et al. (2017) revealed the effectiveness of phage therapy in animals possessing a robust immune system, commonly referred to as ‘immunocompetent’. The innate immune system responds rapidly, and phages operate in collaboration with it to combat infections [122].

Understanding the dynamics between phages, the immune response, and bacterial pathogens is an active area of research. Investigating the specific mechanisms underlying immune recognition and clearance of phages, as well as the modulation of the immune response, can lead to the development of tailored strategies to enhance the efficacy of phage therapy.

### 4.7. Bacteria Classification Based on Their Location in the Host Cells and Their Cell Wall Morphology

It is essential to evaluate whether the efficacy of phage therapy varies based on the type of bacteria involved, such as intracellular versus extracellular bacteria or Gram-negative versus Gram-positive bacteria. These bacterial characteristics can significantly impact phage access, infection dynamics, and the effectiveness of phage therapy, influencing therapeutic outcomes and guiding optimal treatment strategies.

#### 4.7.1. Intracellular Versus Extracellular Bacteria

Phage therapy is typically more effective against extracellular bacteria than intracellular bacteria due to important biological and logistical differences. Extracellular bacteria are accessible to phages since they are found in bodily fluids or on tissue surfaces, where phages can encounter, bind to, and infect bacterial cells. This allows phages to inject their DNA, replicate, and lyse the bacteria, making phage therapy especially useful for treating extracellular infections like wounds or biofilm-associated infections [123].

In contrast, intracellular bacteria reside within host cells, such as macrophages or epithelial cells, where phages cannot reach because they are unable to penetrate eukaryotic cell membranes. This limits the effectiveness of phage therapy against infections where bacteria persist inside host cells. Phages specifically target prokaryotic cells, ensuring that human and animal cells are unaffected—a safety advantage. Research by Kurzepa-Skaradzińska et al. (2013) supports this, showing that bacteriophage preparations do not interfere with the intracellular killing of *E. coli* and *S. aureus* by human phagocytes [124].

Interestingly, phage therapy has shown promise against some intracellular bacteria, including *Mycobacterium tuberculosis* [125], *Mycobacterium abscessus* [126,127], and some Salmonella species [128,129,130].

Phage therapy targeting extracellular bacteria may also synergize with the host immune system to enhance bacterial clearance. However, intracellular bacteria are shielded within host cells, reducing immune accessibility and thus the effectiveness of phage therapy. Recent advancements seek to overcome these challenges, for example, by engineering phages to enter mammalian cells [131,132,133] or by combining phages with antibiotics [120,134] or immune-modulating agents, like antibodies, cytokines, and vaccines [135,136,137], to reduce intracellular bacterial levels.

#### 4.7.2. Gram-Negative Versus Gram-Positive Bacteria

Both Gram-positive and Gram-negative bacteria can be susceptible to phage therapy, but the effectiveness of phage therapy depends largely on factors beyond simply whether a bacterium is Gram-positive or Gram-negative. These factors include the availability of suitable phages, the structural properties of bacterial cell walls, and the phage’s ability to penetrate biofilms or overcome bacterial resistance mechanisms.

Gram-negative bacteria have a complex cell wall with an outer membrane, periplasmic space, and a thin peptidoglycan layer. This outer membrane, containing lipopolysaccharides (LPS), can act as a barrier to phage entry and elicit strong immune responses [138]. In contrast, Gram-positive bacteria lack an outer membrane and have a thick peptidoglycan layer. Phages targeting Gram-negative or Gram-positive bacteria often rely on specific receptors (e.g., LPS, flagella, pili, proteins, and polysaccharides) [139], which can vary across strains and may be modified by bacteria to evade phage infection. Many bacteriophages, especially lytic ones, produce endolysins [140] in combination with other proteins, such as holins, which form pores in the bacterial membrane. Holins allow endolysins to access the peptidoglycan layer, resulting in cell lysis and the release of phage particles.

Phages can also be effective against biofilms, structures that are notoriously resistant to treatment, by lysing bacteria within these protective communities. However, susceptibility to phages varies between Gram-positive and Gram-negative biofilms. For Gram-negative bacteria, biofilms often contain protective extracellular polymeric substances, making them challenging for some phages to penetrate. Certain phages produce depolymerases that can degrade these biofilm components [141], enhancing their effectiveness. Gram-positive bacteria, such as *S. aureus*, also form resilient biofilms, which contribute to their persistence and resistance in various environments, including on medical devices and within human tissues. For example, *S. aureus* bacteriophage SAP-2 contain a cell-wall degrading enzyme (SAL-2), which can be used to prevent and treat biofilm-associated *S. aureus* infections [142]. The formation of biofilms in the Gram-positive bacterium *S. aureus* can vary based on environmental factors, allowing it to adjust its regulatory mechanisms to be either dependent on or independent of polysaccharides [143].

Bacteria can develop resistance to bacteriophages through various antiphage defense mechanisms, posing a significant challenge for phage therapy. These bacterial defenses can disrupt multiple stages of the phage life cycle, including adsorption, DNA injection, genome replication, phage particle assembly, and the release of progeny virions. However, phages exhibit remarkable adaptive flexibility, enabling them to evolve counterstrategies that bypass bacterial defenses, ensuring their survival and continued infectivity. Both phages that target Gram-positive and Gram-negative bacteria have evolved sophisticated mechanisms to overcome bacterial resistance, highlighting their potential as adaptable therapeutic agents [144].

## 5. A Structured Approach to Phage Therapy: From Infection Identification to Treatment Optimization

Phage therapy involves a structured multi-step approach beginning with identifying the infection site and the pathogen’s type and antibiotic susceptibility. This initial assessment helps guide the treatment approach, factoring in the patient’s immune status, whether immunocompetent or immunocompromised. Next, suitable phages are sourced from phage banks, environmental samples, or patient isolates. These phages undergo in vitro testing to confirm their specificity, potential for combination in phage cocktails, and compatibility with adjunctive treatments like Phage-Antibiotic Synergy (PAS).

**Figure 4:**
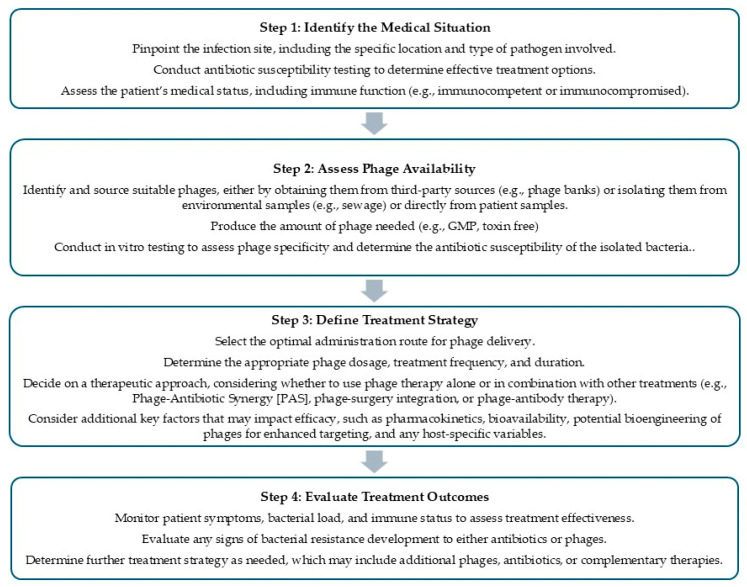
Schematic Overview of Phage Therapy Stages. Illustration of the sequential steps involved in phage therapy, from infection identification to assessment of treatment outcomes.

Once an appropriate phage is identified, the treatment strategy is defined, selecting the optimal administration route, dosage, and frequency. A decision is also made on whether to use phage therapy as a stand-alone treatment or in combination with other therapies, such as antibiotics, surgery, or antibody therapy. Throughout treatment, patient symptoms, bacterial burden, and immune response are monitored to assess efficacy. Any emergence of bacterial resistance to phages or antibiotics is also evaluated to inform further treatment adjustments, which may involve additional phages, antibiotics, or supportive therapies. All these steps are illustrated in Figure 4.

## 6. What Are the Future Perspectives of Phage Therapy?

Phage therapy has the potential to be an important tool for treating bacterial infections, particularly those caused by antibiotic-resistant strains. There are several future perspectives for phage therapy, including the following.

### 6.1. Combination Therapy

Combination therapy, which involves the simultaneous or sequential use of phage therapy alongside traditional antibiotics [145,146] or other treatment modalities, has emerged as a promising method to enhance treatment efficacy and fight the development of bacterial resistance.

In a study conducted by Grygorcewicz et al. (2022), the effectiveness of phage-antibiotic therapies was investigated, revealing diverse interactions between bacteriophages and antibiotics. These interactions were classified into several categories, including synergistic, additive, indifferent, or antagonist, depending on the specific antibiotic employed. The research aimed to elucidate the combined effects of phages and antibiotics, shedding light on their potential for enhanced therapeutic outcomes in the context of bacterial infections [147]. By leveraging the complementary mechanisms of action and synergistic effects, combination therapy offers a multifaceted strategy to address bacterial infections more effectively than single therapies alone. While antibiotics directly target bacteria by interfering with essential cellular processes, phages specifically infect and replicate within bacterial cells, leading to their lysis.

Easwaran et al. (2020) conducted a study to investigate the synergistic effect of phage EcSw (ΦEcSw) in combination with antibiotics against antibiotic-resistant *E. coli*. They found that the combination of ΦEcSw and ampicillin was more effective in inhibiting the antibiotic-resistant *E. coli* strain Sw1 compared to the combination of the bacteriophage with kanamycin or chloramphenicol. It is worth noting that both kanamycin and chloramphenicol inhibited the phage titre, whereas ampicillin did not exhibit phage inhibition. Furthermore, the study demonstrated the clinical relevance of ΦEcSw due to its effectiveness in vivo, as evidenced by the successful retrieval of infected zebrafish and mice [148].

Combination therapy can also help address the issue of resistance development. Bacteria can develop resistance to phages, antibiotics, or both. By employing both phages and antibiotics together, the risk of resistance development can be reduced. The phages can target and eliminate antibiotic-resistant bacteria [149], while the antibiotics can target the non-resistant bacterial population. This approach limits the selective pressure that drives the emergence of resistance, as it becomes more challenging for bacteria to simultaneously develop resistance to both phages and antibiotics. Additionally, the use of combination therapy may enhance bacterial clearance, reduce the duration of treatment, and potentially lower the required antibiotic doses, thereby minimizing the risk of resistance development.

Interestingly, the study of Zhang and colleagues showed that genetic polymorphisms of minor alleles exist in both bacterial and phage genomes. This finding suggests a novel mechanism that enables hosts and phages to rapidly respond to selective pressures from each other. The research focused on *S. aureus* AB91118 and its lytic phage LQ7 as a model system. The study revealed that certain metabolic pathways associated with genes containing unique polymorphic sites could be inhibited by chloramphenicol (CHL). This inhibition resulted in the mutant strain (R1-3-1), resistant to the ancestral phage LQ7, becoming sensitive to this phage. Interestingly, combining CHL with bacteriophages demonstrated reduced resistance and enhanced effectiveness in killing bacteria [150].

Besides antibiotics, using a combination of different treatment approaches can bring additional advantages. For example, combining phage therapy with immune-modulating agents, such as monoclonal antibodies [151,152,153], cytokines [154,155,156], and vaccines [157], can enhance the immune response against bacteria while simultaneously leveraging the bactericidal action of phages. Immune modulation can enhance the recognition and clearance of bacteria by immune cells, leading to improved bacterial eradication. Furthermore, different types of treatments like antimicrobial peptides, biofilm disruptors, or host defense peptides can be used in combination with phages therapy to target specific aspects of bacterial infections, such as biofilm-associated infections, and improve the treatment outcomes.

Recent developments in phage therapy have led to exciting advancements, paving the way for innovative technologies that can enhance the effectiveness of infection treatment. One interesting approach in this field is the creation of APNB, a unique photodynamic antimicrobial agent, as proposed by Ran et al. in their study published in 2021. APNB combines a cationic photosensitizer with bacteriophages, leading to precise elimination of bacteria and demonstrated efficacy against biofilms [158]. Through the utilization of the DNA-binding dye NB, which possesses low systemic toxicity and potential anti-tumoral properties, the combination of selective phototoxicity and phage therapy is achieved. This synergistic approach significantly enhances the overall efficacy of phage therapy, yielding optimal therapeutic outcomes that would otherwise be unattainable.

The design and implementation of combination therapy requires careful consideration of various factors, including the selection of compatible agents, optimal timing, dosing, and the potential for drug interactions. These aspects should be evaluated through preclinical and clinical studies to determine the most effective combinations and treatment protocols for specific bacterial infections.

It is worth noting that combination therapy is not a one-size-fits-all approach and may vary depending on the characteristics of the infection, the bacterial strain involved, and the individual patient’s condition. Personalized medicine approaches, such as tailoring the combination therapy based on bacterial susceptibility testing or patient-specific factors, hold promise for optimizing treatment outcomes.

Braunstein et al. (2024) reported a personalized phage-antibiotic treatment for a Siamese cat suffering from a multidrug-resistant *P. aeruginosa* infection associated with an implant following arthrodesis surgery. The bacteriophage utilized, phage ΦPASB7, had been previously isolated from a water sample collected in Jerusalem in 2022. The treatment regimen combined a personalized anti-*P. aeruginosa* phage with ceftazidime. The phage was applied topically to the surgical wound, while the antibiotic was administered intramuscularly. No side effects were observed during the period of the therapy. After two treatment courses lasting 7 and 3 weeks, respectively, the surgical wound, which had remained open for five months, finally closed completely. The authors of the study noted that they believe this to be the first reported case of personalized phage therapy combined with antibiotics applied to a companion animal [159].

Also, timing is a critical factor in optimizing combination therapies with antibiotics and phages, as these treatments may be applied before, during, or after phage therapy. The interaction between phages and antibiotics in such combinations is complex, influenced by the distinct mechanisms through which antibiotics affect bacterial cells and the specific receptors that phages target on bacterial surfaces. Additionally, the innate immune response [160] and bacterial resistance profiles for both antibiotics and phages play significant roles in the efficacy of these therapies.

In an in vitro study examining *P. aeruginosa* biofilms, researchers found that antibiotic concentration and the timing of administration were key determinants in achieving effective bacterial reduction. Treatments using phages or antibiotics alone had only modest impacts on biofilm reduction. However, a substantial enhancement in bacterial killing was observed when the two were applied simultaneously. Notably, the addition of gentamicin or ciprofloxacin six hours after initial phage treatment resulted in a dramatic biofilm reduction, bringing bacterial counts below detectable levels. This finding underscores the importance of carefully timing and dosing in phage-antibiotic combination therapies to maximize therapeutic outcomes [161]. Similar results were obtained by other investigators [162,163].

### 6.2. Bioengineering Phages

Advances in bioengineering techniques have revolutionized phage therapy, enabling the modification and optimization of phages for enhanced specificity and stability [164,165]. Genetic engineering allows the tailoring of phages’ receptor recognition capabilities to target specific bacterial strains by modifying their receptor-binding proteins or tail fibers [166].

As an example, filamentous phage fd has a predilection for infecting *E. coli* that possesses F pili, whereas filamentous phage IKe targets *E. coli* with N or I pili. In a study conducted by Marzari et al. in 1997, bacteriophages were subjected to genetic modification to broaden their host range and infectivity. They successfully engineered a fusion between the receptor-binding domain of the gene 3 protein (pIII) from IKe phage and the N terminus of the pIII protein from fd phage. This modification effectively expanded the host range of the fd phage. As a result, the engineered fd phage demonstrated the ability to infect *E. coli* strains possessing either N or F pili [167].

Various bacteriophages, such as those from the T2, T4, and T7 families, have been engineered to modify their tail fibers, expanding their host range [168,169,170]. Additionally, other bacteriophages have been optimized to enhance their antimicrobial efficacy by enabling them to deliver biofilm-depolymerases and capsule-depolymerases [171], quorum-quenching enzymes [172], and cell wall hydrolases [173].

Bioengineering also enhances phage stability by genetically engineering resistance to environmental factors [174] and incorporating protective elements. These advancements offer better delivery and prolonged activity at the target site, improving bacterial infection treatment and providing a valuable tool against antibiotic-resistant bacteria and infectious diseases.

Pharmacokinetics, which govern the absorption, distribution, metabolism, and excretion of therapeutic agents, are crucial for effective phage therapy. Bioengineering techniques can be employed to improve the pharmacokinetic properties of phages, such as their circulation time and tissue penetration. Extensive research is underway to enhance the ability of bacteriophages to reach their intended target sites. One key area of focus involves the utilization of encapsulation or entrapment techniques, such as liposomes, fibers, and hydrogels, to facilitate the delivery of phages [175].

For example, in a study conducted by Colom et al. (2015), it was demonstrated that phages of various morphologies can be successfully encapsulated within cationic liposomes. These encapsulated phages exhibited significantly enhanced stability against acidity and lyophilization when compared to their non-encapsulated counterparts in laboratory settings. Moreover, the liposome coating facilitated improved retention of bacteriophages within the chicken intestinal tract and exhibited enhanced effectiveness in eliminating *Salmonella* [176]. In addition, the surface properties of phages can be modified to reduce immunogenicity and enhance their ability to evade immune recognition and clearance. These advancements in pharmacokinetics allow for better control and optimization of phage therapy, maximizing their therapeutic potential.

Moreover, genetic engineering provides the means to introduce genes, proteins, or antimicrobial substances into bacteriophages, thereby augmenting their antimicrobial capabilities. For example, in 2007, Lu and Collins utilized genetic engineering techniques to equip the phage T7 with the enzyme dispersin B (DspB), which is known for its biofilm-degrading properties. Through genetic manipulation, the DspB gene derived from *Actinobacillus actinomycetemcomitans* was integrated into the phage T7 genome under the control of the T7 φ10 promoter. This promoter is recognized by the T7 RNA polymerase. Consequently, the engineered phage T7 exhibited a substantial reduction in bacterial count within a single-species *E. coli* biofilm, surpassing the efficacy of the control T7 phage [171].

In 2010, Pouillot et al. conducted a study using phage engineering to establish phage banks to be used against emerging bacterial strains. Their innovative approach allowed targeted modifications within a gene’s coding sequence, preserving the rest of the gene. By temporarily interrupting the lytic cycle of an obligate virulent phage (T4) and employing homologous recombination, they successfully introduced multiple engineered genes into the genomes of a T4 wild-type phage population. Reactivation of the lytic cycle resulted in the production of engineered infective virulent recombinant progeny. By employing this approach and conducting screenings of these phage banks, they could identify recombinant phage particles with the ability to target bacterial strains distinct from the original ones [177].

It is crucial to ensure that the bioengineered modifications do not compromise the safety and efficacy of phage therapy. Extensive characterization, preclinical studies, and regulatory considerations are necessary to evaluate the functionality, safety, and potential risks associated with the modified phages. Additionally, close collaboration between bioengineers, microbiologists, and clinicians is essential to navigate the challenges and optimize the translation of bioengineered phages into clinical practice.

### 6.3. Bacteriophage Banks

Bacteriophage banks or phage libraries, which involve the construction and utilization of collections of diverse phages, are invaluable resources that can significantly enhance the success of phage therapy. These libraries contain a wide array of phages with diverse host ranges, properties, and genetic characteristics, increasing the likelihood of finding appropriate phages for specific infections [178]. Additionally, phages with unique characteristics can be identified, such as enhanced stability, resistance to environmental stressors, or specific mechanisms to counteract bacterial defense systems.

The construction of bacteriophage banks involves isolating and characterizing phages from various environmental sources, such as soil [179], water [180], or animal microbiota [181]. By sampling different ecological niches, a broad spectrum of phages that have co-evolved with their bacterial hosts can be captured, resulting in a rich diversity of phages with varying properties. The diversity within bacteriophage banks can be further expanded through techniques such as high-throughput sequencing and metagenomics. These methods allow for the identification and characterization of phages directly from environmental samples, providing a comprehensive view of the phage population present in a particular ecosystem. By accessing the genetic information encoded within these phages, researchers can gain insights into their host range, genetic diversity, and potential therapeutic applications.

There are several phage banks available [182] including phage banks from the Israeli Phage Bank (IPB) [183], Eliava Institute of Bacteriophages, Microbiology and Virology of Gorgia [184], Hirszfeld Institute of Immunology and Experimental Therapy in Poland [185], Bacteriophage Bank of Korea [186], and Phage Australia [187].

Phage banks possess several attributes that make them highly suitable for use in the developing world. One key advantage is that the process of isolating, characterizing, and propagating phages is relatively inexpensive. This affordability enables the establishment of phage banks in those regions, even at the local level [182].

Bacteriophage banks also serve as a valuable resource for research and development in phage biology and biotechnology. By systematically characterizing the phages within a library, we can gain insights into phage evolution, genetics, and mechanisms of infection. This knowledge can contribute to our understanding of phage biology and can be harnessed for future advancements in phage therapy, such as the development of phage cocktails [188] or the engineering of phages with desired properties [177].

Furthermore, the availability of bacteriophage banks encourages collaboration and knowledge sharing within the scientific community [183]. Bacteriophage banks can be shared among researchers, facilitating access to a diverse collection of phages, and enabling collaborative efforts to tackle specific bacterial infections. This collaboration promotes the exchange of expertise, resources, and best practices, fostering advancements in phage therapy.

There are two techniques for phage identification in addition to phage banks, one is database-based (alignment-based) methods and the other is non-alignment methods. Database-based methods use a broad collection of sequences as references, while alignment-free methods employ machine learning and deep learning models to detect unique features within sequences [189].

### 6.4. Personalized Medicine

Personalized medicine, an emerging field in healthcare, holds significant promise for the optimization of phage therapy [69,190]. By tailoring phage therapy to individual patients based on their specific bacterial infections and immune responses, personalized approaches can enhance treatment outcomes and improve patient care.

One of the key aspects of personalized phage therapy is the precise identification and characterization of bacterial infection. Through advanced diagnostic techniques, such as whole-genome sequencing or metagenomic analysis, the causative bacterial pathogens can be identified, along with their antibiotic resistance profiles [191,192]. This information is crucial for selecting the most appropriate phages that can effectively target and eliminate the specific bacterial strains causing the infection.

In addition to identifying the infecting bacteria, personalized medicine takes into account the individual patient’s immune response, which plays a vital role in determining the success of phage therapy. By assessing the patient’s immune status, which includes factors such as immune function, antibody levels, and immunogenetic profiles, clinicians can gain insights into how the patient’s immune system is likely to interact with phages. This information helps optimize the process of phage selection and design personalized treatment protocols. For instance, Roach et al. (2017) described the vital synergy between the host immune system and bacteriophage in successfully treating an acute respiratory pathogen through phage therapy [122]. Furthermore, it is important to consider the dynamic nature of bacterial infections and the response of the host immune system over time, allowing adjustments to the treatment as necessary.

Furthermore, personalized medicine approaches can extend beyond the selection of phages. They can encompass other aspects of treatment, such as dosing regimens [193], combination therapies [194], and adjunctive interventions [195]. Factors such as patient characteristics, co-existing medical conditions, and medication interactions can influence the design of personalized treatment protocols. Individualized approaches ensure that the treatment plan is tailored to the specific needs and circumstances of each patient, optimizing the therapeutic benefit and minimizing potential risks.

Implementing personalized phage therapy requires multidisciplinary collaboration between clinicians, microbiologists, immunologists, and bioinformaticians. In their 2022 study, Ferry et al. documented a noteworthy case of a patient suffering from a spinal abscess caused by pandrug-resistant *P. aeruginosa*. The medical team employed a combination of surgical intervention and tailored phage therapy, administered alongside antibiotics. This successful treatment outcome was made possible by a collaborative effort among European academic institutions, dedicated to the discovery, production, and timely delivery of a personalized phage cocktail to the patient. Despite the persistent bacterial presence and the emergence of small colony variants, the patient experienced healing after undergoing two surgeries. The recovery was further supported by the administration of purified phages through local and intravenous injections, serving as an adjuvant therapy [190].

Integrating diverse data sources, such as genomic data, immune profiling, and clinical information, is crucial for making informed decisions and providing personalized care. Moreover, ongoing research and technological advancements play a vital role in enhancing diagnostic tools, treatment algorithms, and our understanding of the intricate interplay between phages, bacteria, and the immune system.

Personalized phage therapy holds significant promise for the future of infectious disease treatment. By customizing treatment strategies for individual patients, it offers the potential for higher success rates, reduced adverse effects, and improved patient outcomes. However, it is important to acknowledge that implementing personalized medicine approaches in phage therapy is still in its early stages. Further research and clinical validation are necessary to establish best practices and refine treatment protocols.

### 6.5. Phage Cocktail

Phage therapy holds promising future potential through the utilization of phage cocktails. These cocktails offer enhanced targeting capabilities against a broader range of bacteria, while also addressing resistance issues through diverse mechanisms of action. Additionally, the synergistic effects achieved by combining multiple phages can further enhance therapeutic outcomes. By harnessing the power of phage cocktails, the field of phage therapy has the potential to revolutionize the treatment of bacterial infections. Tailored treatments based on personalized patient profiles and the combination of phages with antibiotics hold promise. Advancements in delivery systems, like nanotechnology, could improve precision and efficiency. These developments have the potential to greatly enhance phage therapy’s effectiveness and applicability in combating bacterial infections.

A study published in Frontiers in Microbiology highlights the absence of a universally accepted “gold standard” for developing a phage cocktail. However, the study introduces a novel approach to creating an effective phage cocktail specifically targeting ESBL-producing *E. coli* and *Klebsiella* strains commonly found in UK hospitals [196]. The researchers tested the phage cocktail by introducing selected phages to a combination of seven *E. coli* isolates. The choice of host strains was based on their distinct proteomic profiles, which exhibited a moderate positive correlation with their sensitivity to the phages [197].

Another study published in MDPI explores the rational design of phage cocktails for phage therapy. The research aims to maximize the impact on a broader range of bacteria while minimizing the likelihood of a subset of those bacteria developing phage resistance. This approach is based on leveraging previously identified phage properties to inform the design process [198].

Phage cocktail therapies have demonstrated significant potential in treating multidrug-resistant (MDR) infections across diverse clinical scenarios. Several key studies underscore their effectiveness and safety profile in human applications. In a randomized placebo-controlled trial by Karn et al. (2024), phage therapy was evaluated for chronic wound infections caused by MDR bacteria. Patients treated with the bacteriophage cocktail achieved a 93.3% sterility rate within 39 days and complete wound healing by day 90, contrasting with continued bacterial colonization in the placebo group [16]. Similarly, Samaee et al. (2023) conducted a double-blind trial using an inhaled phage cocktail targeting *P. aeruginosa*, Acinetobacter, and MRSA in COVID-19 patients with secondary bacterial pneumonia. The treatment led to faster symptom resolution and negative sputum cultures, although there were no significant differences in ICU stay or survival rates. The therapy was well-tolerated, and further trials were recommended [17].

In another instance, Terwilliger et al. (2021) reported the successful use of a phage cocktail in an immunosuppressed liver transplant patient suffering from recurrent urinary tract infections (UTIs) due to extended-spectrum beta-lactamase-producing *E. coli*. Combined with ertapenem, the phage therapy resulted in negative urine cultures and a symptom-free follow-up, highlighting its safety and efficacy in a vulnerable patient population [18].

Furthermore, Kim et al. (2024) described the ELIMINATE phase 2 trial, which tested a CRISPR-Cas3-enhanced phage cocktail, LBPEC01, for treating drug-resistant uncomplicated UTIs. Patients receiving intraurethral and intravenous LBPEC01 treatment experienced rapid bacterial reduction, were symptom-free by day 10, and showed no adverse effects [19].

Lastly, Qi et al. (2021) presented a case in which a phage cocktail successfully eradicated MDR *K. pneumoniae* in a patient’s urinary tract after antibiotics had failed. The phage mixtures were refined to optimize lytic activity, leading to complete bacterial clearance and improved bladder health [20].

Collectively, these studies underscore the therapeutic potential of phage cocktails as viable alternatives or adjuncts to antibiotics in treating MDR infections, particularly in complex and otherwise intractable cases.

### 6.6. Environmental Phage Therapy

Environmental phage therapy, an emerging field with diverse applications, holds significant potential for controlling bacterial populations and addressing challenges in various environmental settings. This approach utilizes phage therapy to combat bacterial pathogens in areas such as agriculture [199], food safety [200], and water treatment [201], offering innovative and targeted solutions.

In agriculture, phage therapy presents a promising alternative to traditional pesticides and antibiotics [199] for managing bacterial diseases in crops and livestock. Bacterial pathogens can cause significant damage to agricultural production, leading to economic losses and environmental concerns. By identifying and employing phages that specifically target these pathogens, it is possible to mitigate their impact on crops and livestock. Phages can be applied through sprays, irrigation systems, or biocontrol agents, selectively reducing the target bacterial populations without affecting beneficial organisms. This environmentally friendly approach minimizes the use of chemical agents and contributes to sustainable agriculture practices.

Food safety [200] is another area where environmental phage therapy can play a vital role. Bacterial contamination of food products can lead to foodborne illnesses and outbreaks. Traditional methods of disinfection, such as chemical treatments or heat processing, may have limitations and can impact the sensory and nutritional quality of food. Phage therapy offers a targeted and precise approach to control bacterial pathogens in food. Phages can be used as biocontrol agents to selectively eliminate harmful bacteria, such as *Salmonella* or *E. coli*, reducing the risk of foodborne infections. This targeted intervention can enhance food safety measures and reduce the reliance on chemical disinfectants. For example, El-Gohary et al. (2014) demonstrated the practicality and effectiveness of enhancing the environment with bacteriophages as a preventive measure against colibacillosis in broiler chickens. Their research showcased the positive outcomes achieved through the augmentation of the chicken environment with bacteriophages. By utilizing this approach, the incidence of colibacillosis, a bacterial infection caused by *E. coli*, was effectively reduced. Aerosol sprays with bacteriophages administered to both poultry and bedding materials have proven effective in this regard. These advancements not only highlight the potential of phage therapy in addressing antimicrobial resistance but also demonstrate its practical applications in different domains, such as agriculture and food safety [202].

Phage therapy also has applications in water treatment [201] where bacterial contamination poses a significant public health concern. Waterborne pathogens, including those resistant to conventional disinfection methods, can lead to waterborne diseases and outbreaks. Environmental phage therapy provides a potential solution by using phages to target and eliminate specific bacterial pathogens present in water sources. Phage-based treatments can be integrated into water treatment systems, such as filtration or disinfection processes, to enhance the removal of bacterial contaminants. This approach offers a complementary tool to traditional water treatment methods, contributing to improved water quality and public health.

To effectively apply environmental phage therapy, several factors need to be considered. Firstly, the selection of phages should be based on thorough characterization and understanding of the target bacteria and their specific environmental conditions. Phage cocktails or combinations may be necessary to address the diversity of bacterial populations and their potential resistance mechanisms. We must also be aware of the potential dangers of phage-mediated horizontal gene transfer among pathogenic and non-pathogenic bacterial species [203].

Additionally, regulatory considerations, safety assessments, and monitoring protocols should be in place to ensure the safe and responsible use of phages in environmental settings.

Ongoing research and development are essential to advancing environmental phage therapy. This includes the discovery and characterization of new phages, optimization of delivery methods, development of phage formulations suitable for environmental applications, and assessment of their ecological impact. Collaboration between researchers, regulatory agencies, and stakeholders in agriculture, food production, and water management is crucial to drive innovation, address challenges, and ensure the successful implementation of environmental phage therapy.

### 6.7. Applications of Phage Therapy in Non-Infectious Diseases

Bacteriophages, or phages, have emerged as promising tools beyond their traditional role in combating bacterial infections, showing potential in the treatment and prevention of non-infectious diseases. Their unique properties have paved the way for applications in cancer therapy [204], immune modulation [205], and innovative vaccine development [10,11].

In cancer therapy, phage display technology has allowed for the precise targeting of cancer markers by selecting peptides or antibodies that bind specifically to tumor cells. This enables the delivery of therapeutic agents directly to cancer cells, minimizing damage to healthy tissues and enhancing the effectiveness of anticancer drugs. Phages are also being explored as “oncolytic” agents; while traditional oncolytic viruses directly kill cancer cells, engineered bacteriophages can be used to deliver genes or molecules that promote cancer cell death or inhibit tumor growth [206]. For example, they can serve as vectors for CRISPR-Cas9, allowing targeted inactivation of cancer-promoting genes, thus adding a powerful tool to precision medicine in oncology [207].

In inflammatory conditions, bacteriophages demonstrate significant immunomodulatory potential [208]. Through their natural interactions with immune cells, phages can be engineered to display anti-inflammatory peptides [209] or therapeutic proteins, offering a targeted approach to reduce inflammation in chronic diseases like rheumatoid arthritis and inflammatory bowel disease. Additionally, phages indirectly contribute to anti-inflammatory therapies by selectively targeting and reducing pathogenic bacteria that drive inflammation and by modulating the microbiome to alleviate systemic inflammatory responses. This combined approach not only helps restore immune balance but also promotes tissue healing in affected areas.

Phage-based vaccines represent another promising area, where phages can act as carriers for antigens [210,211]. By displaying viral or tumor antigens on their surfaces, phages can stimulate the immune system without the need for a live virus. This capability is particularly valuable in cancer immunotherapy, where a tailored immune response against tumor antigens could serve as a form of personalized cancer vaccine. Additionally, phages have inherent adjuvant properties that can enhance the immune response to the antigens they carry, making them useful for vaccines against cancer and other diseases that require a robust immune response.

These innovative applications of phages highlight their versatility—not only as therapeutic agents but also as delivery systems for bioactive molecules. This expands their potential for managing non-infectious diseases and advancing preventative medicine, opening new pathways in healthcare.

### 6.8. Phage Dosing

Phage dosing is a critical factor that can significantly influence the outcomes of phage therapy in the treatment of infectious diseases. Here are several key aspects regarding how dosing affects therapy effectiveness.

#### 6.8.1. Inoculum Size

The initial bacterial burden or inoculum size plays a crucial role in determining the appropriate phage dose. A higher bacterial burden may require a higher phage dose to effectively establish an infection and ensure sufficient phage–bacteria interactions. Insufficient dosing may lead to inadequate bacterial lysis, allowing the infection to persist.

Delattre et al. (2022) studied the dynamics of phage–bacteria interactions in vivo context using a mathematical model and found that the initial bacterial burden was the most critical factor. In animals with a starting bacterial inoculum of less than 6 log10 CFUeq/g, the bacteria quickly dropped below detectable levels within 48 h, regardless of the treatment used. However, in animals with a high initial bacterial inoculum (greater than 8 log10 CFUeq/g), phage therapy alone could not control the bacteria’s continued growth, no matter the administration route or phage dose. The benefit of phage therapy was most noticeable in animals with an intermediate initial bacterial inoculum, between 6 and 8 log10 CFUeq/g [212].

#### 6.8.2. Multiplicity of Infection (MOI)

The ratio of phages to bacteria, known as the multiplicity of infection, is essential in phage therapy. An optimal MOI can enhance phage efficacy by ensuring that enough phages are available to bind to and infect the bacteria. However, if the MOI is too low, phages may not successfully compete with the bacterial population, leading to treatment failure. Determining the MOI in an in vivo setting can be challenging, as bacterial levels often fluctuate between the initial infection and the time of phage administration, making it difficult to accurately measure bacterial density just before treatment. Moreover, targeted bacterial populations may not be uniformly accessible to phages, as some bacteria may reside in locations that limit phage penetration and efficacy [213].

#### 6.8.3. Phage Kinetics

The dynamics of phage replication and bacterial lysis are influenced by the dosing regimen. Higher doses can lead to a rapid increase in phage concentration, which can overwhelm bacterial defenses and enhance lysis. The initial number of administered phage particles serves as the basis for determining phage kinetics [97]. Suboptimal dosing can lead to slower kinetics, giving bacteria more time to evade phage activity.

#### 6.8.4. Phage Resistance Development

During phage therapy, bacterial resistance can emerge [214] if dosing is insufficient. Low phage concentrations may allow some bacteria to survive and develop resistance mechanisms, potentially reducing the effectiveness of future treatments. Ensuring adequate dosing helps minimize resistance by effectively targeting and lysing the majority of bacterial cells.

#### 6.8.5. Therapeutic Window

Determining the right dose is essential for establishing a therapeutic window—an effective dose that maximizes therapeutic benefits while minimizing potential side effects [215]. High doses may lead to adverse reactions or toxicity, especially if phages are delivered in conjunction with other treatments, such as antibiotics.

#### 6.8.6. Timing and Frequency of Dosing

The timing and frequency of phage administration can also impact treatment outcomes. Administering multiple doses over a defined schedule can maintain therapeutic phage levels in the body, enhancing efficacy. A continuous or pulsatile dosing strategy may optimize the interaction between phages and bacteria, leading to improved treatment success.

#### 6.8.7. Patient-Specific Factors

Individual patient factors, including immune status, underlying health conditions, and microbiome composition, can influence how phages interact with bacterial infections. Personalized dosing strategies based on these factors may improve the effectiveness of phage therapy.

## 7. Is Phage Therapy Regulated?

While phage therapy has yet to receive approval for general use in the United States or Europe, its significant potential has gained global recognition. However, regulatory support for commercial applications is still limited. In the United States, the Food and Drug Administration (FDA) plays an active role in evaluating the feasibility of phage therapy as an investigational treatment. Programs like Expanded Access or Compassionate use [216] and Clinical Trials pave the way for experimental therapies, offering a lifeline to patients battling serious or life-threatening infections when conventional options have been exhausted. Physicians can advocate for their patients, seeking access to these treatments, while the FDA conducts thorough case-by-case evaluations. Key considerations include the patient’s condition, available clinical data, and a meticulous assessment of the potential risks and benefits. It is important to note that participation in expanded access programs does not automatically guarantee widespread approval. Nevertheless, the FDA’s rigorous evaluation and approval processes remain crucial for phage therapy to be officially recognized as a standard treatment option in the future.

In some European countries, phage therapy has been permitted on a case-by-case basis or within clinical trials. The European Medicines Agency (EMA) have classified phage therapies as novel under Regulation (EU) 2019/6, which require marketing authorization through a centralized procedure. The Novel Therapies and Technologies Working Party (NTWP) has developed a guideline focusing on quality, safety, and efficacy requirements to establish a regulatory framework for bacteriophage products and encourage their development [217].

Poland has a rich history of research and clinical use of phage therapy, with the Phage Therapy Unit at the Ludwik Hirszfeld Institute of Immunology and Experimental Therapy in Wrocław at the forefront. The Laboratory of Bacteriophages at the Polish Academy of Sciences utilize phage therapy to treat patients with antibiotic-resistant bacterial infections. Phage therapy is currently being explored in Poland and has received some regulatory approvals for experimental and compassionate use. However, it is not yet considered part of mainstream medicine. The Eliava Institute of Bacteriophage, Microbiology, and Virology in Tbilisi, Georgia, is a leading center for phage therapy research and production [218]. Phage therapy is officially recognized and regulated in Russia, with multiple phage therapy centers across the country providing treatment for various bacterial infections [219]. In addition to the pharmaceutical applications of phage therapy in Poland and Russia, the FDA has authorized certain commercial phage formulations for use in the food industry to control specific pathogenic bacteria [220].

## 8. Discussion

This study stands out from previous reviews by offering a comprehensive multifaceted analysis of phage therapy as a therapeutic option, addressing aspects of safety, preclinical and clinical efficacy, and regulatory perspectives. While many reviews have focused on isolated components of phage therapy, our study synthesizes insights from a wide range of research. Additionally, this review explores critical factors that influence treatment efficacy—such as infection site, phage–host specificity, bacterial load, phage pharmacokinetics (including administration route), patient immune responses, bacterial location within host cells, and cell wall morphology—highlighting the importance of these considerations in optimizing therapeutic outcomes.

To facilitate practical application, we developed a streamlined four-step guideline for phage therapy, providing a structured framework to guide practitioners from infection identification through treatment planning. Future directions identified in our review include combination therapies, bioengineered phages, bacteriophage banks, personalized medicine, phage cocktails, environmental phage therapy, applications in non-infectious diseases, and optimized phage dosing strategies.

By addressing the challenges and regulatory landscapes across Western and Eastern contexts, this review provides a holistic view of phage therapy’s potential and limitations, underscoring the need for rigorous clinical trial data to promote regulatory acceptance and its eventual integration into mainstream medicine.

Phage therapy has demonstrated a good safety profile in early studies and clinical trials, with minimal adverse effects. Its high specificity in targeting specific bacteria while sparing beneficial ones contributes to its safety. Safety evaluations have been conducted through various administration routes, including oral, local, intravenous, and inhalation. Both preclinical studies in animals and clinical trials in humans have confirmed the safety of phage therapy, with observed adverse effects generally being mild and temporary. For instance, local reactions, transient flu-like symptoms, and mild discomfort have been reported. Clinical trials specifically assessing the safety of orally administered phages and phages in mineral water have shown no significant adverse effects and good tolerability. These findings collectively demonstrate the safety of phage therapy as a potential treatment option for bacterial infections. So, we can conclude that phage therapy seems to be safe. However, before any new phage can be used in in vivo studies, it is essential to conduct thorough in vitro analyses. These analyses should not only assess the phage’s specificity for its target bacterial host but also examine its genome for the presence of any undesirable genes. Specifically, the phage’s DNA must be screened for antibiotic resistance genes or virulence factors that could have been acquired from pathogenic bacteria. Ensuring the absence of these harmful genetic elements is crucial to prevent the potential spread of antibiotic resistance or exacerbation of pathogenicity during phage therapy.

The efficacy of phage therapy has been well-documented in a variety of preclinical and clinical studies targeting different types of infections. For instance, in wound infections caused by *K. pneumoniae, P. aeruginosa*, and *S. aureus*, phage therapy has demonstrated enhanced wound healing, effective elimination of the targeted bacteria, and reduced infection rates. In pneumonia models, phage therapy has shown promise against methicillin-resistant *S. aureus* (MRSA) and *K. pneumoniae*, preventing severe pathological lesions and significantly reducing bacterial loads in the lungs.

Moreover, phage therapy has proven effective in treating urinary tract infections (UTIs) caused by uropathogenic *E. coli*, leading to bacterial eradication and symptom relief. In gastrointestinal infections, phage therapy successfully reduced bacterial burdens and prevented clinical symptoms caused by pathogens such as *C. difficile*, *V. cholerae*, and *Salmonella* spp. Studies conducted in bacteremia models involving *E. faecium*, *P. aeruginosa*, *K. oxytoca*, and *K. pneumoniae* have demonstrated the rescue of animals from fatal bacteremia and improved survival rates through phage therapy.

Clinical trials have also shown promising results in treating burn wound infections, cholera, enterotoxigenic and enteropathogenic *E. coli* diarrhea, and UTIs, with outcomes such as bacterial clearance, clinical improvement, and symptom reduction.

However, it is important to acknowledge that not all studies have demonstrated the efficacy of phage therapy. In some cases, this could be attributed to suboptimal conditions, such as an insufficient multiplicity of infection (MOI) or the presence of natural barriers that impede the phages’ ability to effectively reach and target the bacteria. Therefore, optimizing these conditions and thoroughly investigating various factors are essential to achieving positive outcomes with phage therapy before advancing to in vivo studies.

In addition to the application of phage therapy in bacterial infections, recent studies have highlighted the therapeutic potential of bacteriophages in the context of COVID-19, particularly in combatting SARS-CoV-2. Research in a hamster model of SARS-CoV-2 infection revealed both preventive and therapeutic effects of phage treatment, with treated hamsters showing reduced viral loads and milder clinical symptoms, potentially due to enhanced immune responses against the virus [221]. In addition, it has been suggested that bacteriophages can be used as anti-inflammatory agents to control the cytokine storm in SARS-CoV-2 infections [222].

Numerous factors can influence the efficacy of phage therapy, making it essential to address these variables to achieve successful treatment outcomes. One of the most critical factors is phage–host specificity, which requires the careful identification and matching of appropriate phages to effectively target and combat bacterial infections. Additionally, the bacterial burden and the ratio of bacterial inoculum to phage inoculum are crucial, as a significant number of phages may be lost or cleared by the body’s natural barriers, resulting in only a fraction of the phages reaching the target site. Once at the site, phages face further challenges, such as bacterial biofilms, bacterial diversity within the population, and the development of phage resistance.

Understanding the pharmacokinetics of phage therapy, including factors such as the route of administration, dosage, frequency of treatment, metabolism, and elimination, is also vital to the overall effectiveness of the therapy. The host immune response can further impact the efficacy of phage therapy, necessitating strategies to optimize therapeutic outcomes by considering factors like immunogenicity and immune evasion.

To enhance the efficacy and precision of phage therapy, several advanced strategies should be explored. These include the use of combination therapies, bioengineering phages to enhance their effectiveness, accessing established phage banks for a broader range of options, and employing phage cocktails to simultaneously target multiple bacterial strains. Additionally, adopting personalized medicine approaches tailored to the specific needs of individual patients can further optimize treatment outcomes. By addressing these factors and integrating these innovative strategies, the success of phage therapy can be significantly improved.

Although phage therapy is not yet widely approved in the United States and Europe, there are pathways for its evaluation. In the U.S., the FDA assesses phage therapy through programs like Expanded Access and Clinical Trials, but these pathways do not guarantee approval. In Europe, phage therapy is allowed on a case-by-case basis or within the framework of clinical trials, requiring authorization from the EMA. In countries such as Poland, Georgia, and Russia, phage therapy has gained more recognition, but detailed information regarding their regulatory frameworks remains limited.

## 9. Conclusions

Phage therapy is a powerful therapeutic option for combating bacterial infections, particularly those caused by antibiotic-resistant strains and in situations where all other treatments have been exhausted. Its unique ability to specifically target and eliminate bacteria without causing harm to humans or animals makes it a highly promising alternative to traditional antibiotics. Extensive evidence supports the efficacy of phage therapy in treating a variety of infections, even though some studies have not consistently demonstrated its effectiveness. Notably, numerous case reports have documented the successful use of phage therapy in emergency situations, often saving patients’ lives when no other options were available.

Despite its potential, phage therapy faces significant barriers to widespread recognition and approval, particularly in the U.S. and Europe. Regulatory approval remains limited in these regions, hindering its integration into mainstream medical practice. In contrast, countries like Russia have a long history of using phage therapy, particularly for treating wounds, where it has been employed for decades with notable success. To overcome these barriers, there is a pressing need for increased efforts from researchers and therapeutic sponsors to generate robust data from clinical trials. These data are essential for convincing regulatory agencies and clinicians of the safety and efficacy of phage therapy. Achieving positive outcomes requires careful consideration of all factors that influence phage therapy, including phage–host specificity, dosing, and the challenges posed by bacterial biofilms and resistance.

Additionally, phage therapy holds great promise as an accessible therapeutic option for developing countries. Phages are relatively easy to produce and can provide a rapid and cost-effective solution to bacterial infections, even in remote areas where traditional medical resources may be scarce. This makes phage therapy not only a viable treatment option in developed nations but also a critical tool in global health efforts to address bacterial infections in underserved populations. By addressing the current challenges and leveraging the potential of phage therapy, it can become a cornerstone in the fight against bacterial infections worldwide.

## Figures and Tables

**Table 4:** Phage therapy for urinary tract infections.

Phage	Origin	Challenge Organism	Bacterial Inoculum (CFU)	Phage Inoculum (PFU)	Delivery Method	Treatment Schedule	Type of Model	Outcome	Reference
PEC80 or a phage cocktail (PEC3, PEC11, PEC15, PEC16, PEC28, PEC30, PEC36, PEC37, PEC38, PEC44, PEC51, PEC52, PEC55, PEC63, PEC68, PEC78, PEC80, PEC94, PEC102, PEC133, PEC215, PEC301, PEC304, PEC305, and PEC306)	Unknown	Uropathogenic *E. coli*	10^6^	10^6^	Transurethrally or i.p.	10 days post-challenge	Mouse	PEC80 alone did not affect the therapy, but both delivery approaches of the cocktail formulation resulted in bacterial eradication	Mijbel Ali et al. (2021) [73]
Cocktail (HP3, HP3.1, ES17, and ES19)	Sewage and Goose/Duck Feces	*E. coli*	N/A	10^9^ PFUs/mL	i.v.	Every 12 h	Human, case report	No bacteria were detected in the urine after the first dose of the phage and ertapenem.	Terwilliger et al. (2021) [18]
Cocktail LBP-EC01	Engineered with a CRISPR-Cas3 construct targeting the *E. coli* genome	*E. coli*	N/A	200 mL of 2 × 10^12^ PFU LBP-EC01, alongside oral trimethoprim/sulfamethoxazole	i.u. administration via catheters and i.v.	2 and 3 days	Phase 2 clinical trial, 39 female patients with uncomplicated urinary tract infections (uUTIs)	Rapid reduction of *E. coli* in urine on Day 10, and free of UTI symptoms on Day 10 as well as on Day 34	Kim et al. (2024) [19]
Cocktails (Ф902, ФJD905, ФJD907, ФJD908, and ФJD910)	Collected from a diverse range of environmental samples	Multidrug-resistant *K. pneumoniae* UTI	N/A	2.5 × 10^10^ via bladder and 5 × 10^9^ via the kidney	Irrigation of the bladder and kidney	Every 48 h for 2 weeks	Human, case report,	Alleviated the infection symptoms and successfully eradicated the bacteria from the patient’s urine.	Qi et al. (2021) [20]

CFU, Colony-forming unit; PFU, Plaque-forming units; N/A, Not applicable; i.p., Intraperitoneal; i.u., Intrauterine; i.v., Intravenous.

## Data Availability

Not applicable.
